# The dynamic trajectory of circulating exosome components in high-fat diet-induced metabolic-associated steatotic liver disease and its modulation by curcuminoids

**DOI:** 10.3389/fendo.2026.1933356

**Published:** 2026-09-11

**Authors:** Xingke Zhu, Xiaoliu Hu, Zecai Zhan, Zhaoxiang Zeng, Xueyan Zhao, Hanmin Li, Chengwu Song, Bo Li, Shuna Jin

**Affiliations:** 1 School of Pharmacy, Hubei University of Chinese Medicine, Wuhan, Hubei, China; 2 Huanggang Industrial Injury Insurance Service Center, Huanggang, Hubei, China; 3 Hubei Shizhen Laboratory, Wuhan, China; 4 Institute of Liver Disease, Hubei Provincial Hospital of Traditional Chinese Medicine, Affiliated Hospital of Hubei University of Chinese Medicine, Wuhan, China; 5 Hubei Key Laboratory of Theory and Application Research of Liver and Kidney in Traditional Chinese Medicine, Wuhan, China; 6 Department of Laboratory Medicine, Hubei Provincial Hospital of Traditional Chinese Medicine, Affiliated Hospital of Hubei University of Chinese Medicine, 856 Luoyu Road, Hongshan District, Wuhan, China; 7 School of Basic Medical Sciences, Hubei University of Chinese Medicine, Wuhan, Hubei, China

**Keywords:** circulating exosomes, curcuminoids, MASLD, nanoparticle tracking analysis, temporal clustering analysis, untargeted metabolomics

## Abstract

**Background and objective:**

Circulating exosomes have emerged as promising non-invasive carriers of disease biomarkers, particularly in metabolic-associated steatotic liver disease (MASLD). Curcuminoids derived from *Curcuma longa L.* have shown potential in regulating lipid metabolism. However, most studies focus on late-stage disease, overlooking the dynamic metabolic changes during early progression, and the effect of curcuminoids on circulating exosomal components remains unknown. This study aimed to characterize the dynamic trajectory of circulating exosome components in high-fat diet (HFD)-induced MASLD and to evaluate its modulation by curcuminoids.

**Methods:**

A time-course mouse model of HFD-induced MASLD was established over 12 weeks. Untargeted metabolomics combined with temporal clustering analysis and nanoparticle tracking analysis was used to profile dynamic changes in circulating exosomal lipids and identify candidate biomarkers. Subsequently, the curcuminoids were administered preventively to HFD-fed mice, and targeted metabolomic analysis of the selected biomarkers was performed to assess intervention-induced alterations and their correlation with exosome particle concentration.

**Results:**

Seventy-one time-independent differential metabolites were identified between HFD and control groups. Temporal clustering analysis revealed distinct dynamic patterns of 43 metabolites between MASLD and control mice. Correlation analysis with exosome secretion dynamics identified 36 metabolites significantly associated with exosomal particle concentration, among which 26 were ultimately selected as robust lipid biomarkers. The curcuminoids significantly ameliorated MASLD-related phenotypes, including serum lipids, liver enzymes, hepatic steatosis, and brown adipose tissue injury. Quantitative analysis further showed that 22 of the 26 biomarkers were significantly downregulated by curcuminoids, with these changes correlating with exosomal particle concentration.

**Conclusion:**

This study provides a comprehensive temporal landscape of exosomal metabolite changes during MASLD progression and demonstrates that curcuminoids intervention is associated with modulation of disease progression, paralleled by downregulation of exosome-associated lipid metabolites. The identified metabolites represent potential early-stage, non-invasive biomarkers for MASLD that warrant further validation in human cohorts and offer preliminary experimental evidence for the effects of the curcuminoids in MASLD.

## 1 Introduction

Metabolic-associated steatotic liver disease (MASLD) represents the most prevalent chronic hepatic disorder and constitutes a public health burden, affecting approximately one-third of the general adult population globally (1, 2). MASLD represents a disease spectrum that encompasses various stages ranging from simple hepatic steatosis to hepatocellular carcinoma (3). Early diagnosis of MASLD is critical in reducing the risk of long-term adverse outcomes for patients. For a long time, liver biopsy has been the definitive reference standard for diagnosing MASLD (4). Given the invasiveness of liver biopsy, which is not suitable for routine screening or continuous monitoring of disease progression, non-invasive detection methods have been proposed for the diagnosis of MASLD (5–7). Although imaging techniques and serum-based detection methods have been explored, their reliability in differentiating the progression of MASLD remains limited (8–11). Therefore, the development of non-invasive and highly accurate diagnostic tools represents a priority for improving early detection and longitudinal monitoring of MASLD.

Among emerging non-invasive approaches, circulating exosomes have garnered particular interest due to their ability to reflect disease-associated molecular alterations. Circulating exosomes are extracellular vesicles with diameters ranging from 30 to 150 nanometers, carrying molecular “snapshots” of the molecules from the source cells, including proteins, lipids, and nucleic acids (12, 13). They have been increasingly recognized as a rich source of non-invasive, disease-specific biomarkers (14). The strong correlation between the molecular composition of circulating exosomes and hepatic metabolic dysregulation and inflammation has prompted intensified research efforts to explore their value as potential biomarkers for MASLD and related liver pathologies (15, 16). However, most studies have focused on analyzing exosomal components in the context of advanced or end-stage liver disease (17–19). This static perspective overlooks the dynamic metabolic and signaling changes that occur during the initiation and progression of MASLD, thereby limiting the potential of exosomes for early diagnosis and real-time monitoring of disease progression. Therefore, elucidating the dynamic changes in circulating exosomal biomarkers throughout the course of MASLD may provide critical insights into its pathogenesis and offer a theoretical basis for early diagnosis and therapeutic intervention.

Curcuminoids, the major bioactive polyphenols derived from *Curcuma longa L.*, are well recognized for their multiple pharmacological activities, including anti-inflammatory, antioxidant, and lipid-regulating properties (20–22). In MASLD, curcuminoids have been shown to improve liver function and attenuate disease progression in both preclinical models and clinical studies (23, 24). Despite these well-documented benefits, whether curcuminoids exert their protective effects through modulation of circulating exosomal components remains unclear. Given the emerging role of exosomes in inter-organ communication and lipid signal transmission, investigating the impact of curcuminoids on the dynamic trajectory of exosomal lipids during MASLD progression warrants further investigation.

In this study, an animal model of MASLD was established and assessed at five time points to monitor disease progression alongside dynamic changes in circulating exosomes. Untargeted metabolomics and temporal clustering analysis were subsequently applied to characterize the dynamic alteration patterns of metabolites in circulating exosomes throughout MASLD development. Finally, nanoparticle tracking analysis and correlation analysis were employed to identify circulating exosomal biomarkers capable of accurately reflecting the progression of MASLD. Subsequently, the curcuminoids were administered preventively to high-fat diet (HFD)-fed mice, and quantitative analysis of the selected biomarkers was performed to evaluate its modulatory effects. This study offers novel insights and promising tools for the early detection and monitoring of MASLD, while also providing experimental evidence for the metabolic effects of the curcuminoids in MASLD.

## 2 Methods

### 2.1 Chemicals and materials

HPLC-grade acetonitrile and methanol were procured from Fisher Scientific (Fair Lawn, NJ, USA), while deionized water was generated in-house utilizing a Milli-Q purification apparatus (Millipore, Bedford, MA, USA). Commercial assay kits for total cholesterol (TC; Cat. No. A111-1-1), triglycerides (TG; Cat. No. A110-1-1), high-density lipoprotein cholesterol (HDL-C; Cat. No. A112-1-1), and low-density lipoprotein cholesterol (LDL-C; Cat. No. A113-1-1) were obtained from Nanjing Jiancheng Bioengineering Institute (Nanjing, China). Kits for alanine aminotransferase (ALT; Cat. No. E-BC-K235-M) and aspartate aminotransferase (AST; Cat. No. E-BC-K236-M) were purchased from Elabscience Biotechnology Co., Ltd. (Wuhan, China). Shanghai YuanYe Bio-Technology Co., Ltd. (Shanghai, China) supplied phosphotungstic acid (Cat. No. R20745; 100 mL). Antibodies used in this study included CD9 polyclonal antibody (Cat. No. 20597-1-AP) and calnexin monoclonal antibody (Cat. No. 66903-1-Ig) from Wuhan Sanying Biotechnology Co., Ltd. (Wuhan, China), and anti-TSG101 antibody (Cat. No. GB15619) from Wuhan Seville Bioengineering Co., Ltd. (Wuhan, China). *Curcuma longa L.* rhizomes were purchased from Gaoyao City, Guangdong Province, China, and authenticated by the Department of Pharmacognosy, Hubei University of Chinese Medicine. Curcuminoids used in this study were in-house enriched and prepared (25). The enriched fraction contained eight curcuminoids, including curcumin, demethoxycurcumin, bisdemethoxycurcumin, and five dihydro- and tetrahydro-curcuminoids, with a purity exceeding 70% (25).

### 2.2 Model establishment

Six-week-old male C57BL/6J SPF mice (license No. SYXK [E] 2023-0067) were obtained from Hunan Slack Jingda Laboratory Animal Co., Ltd. (Wuhan, China). The Institutional Animal Care and Use Committee of Hubei University of Chinese Medicine approved all animal procedures. Mice were housed under specific pathogen-free (SPF) conditions at 24 ± 2 °C and 60 ± 5% relative humidity, under a 12-hour light-dark schedule, with unrestricted access to ordinary chow and water.

After 5 days of acclimatization, 99 mice were divided randomly into nine groups (n = 11 per group): baseline (week 0, normal diet (ND)-HFD-0), ND-fed groups at weeks 3, 6, 9, and 12 (ND-3, ND-6, ND-9, ND-12), and HFD-fed groups at the same time points (HFD-3, HFD-6, HFD-9, HFD-12). For the 12-week feeding regimen, control groups received standard chow, whereas HFD groups were fed a custom high-fat diet composed of 67.8% standard chow, 15% egg yolk powder, 15% lard, 2% cholesterol, and 0.2% sodium cholate. Nutritional composition, as analyzed by Pu-Ni Testing Group Co., Ltd., confirmed a fat content of 31.2% (w/w), accounting for approximately 54% of total caloric intake. Samples were collected at weeks 0, 3, 6, 9, and 12. Serum samples were used for biochemical assays and circulating exosome isolation. Livers were excised, rinsed with cold saline, weighed, and subjected to Oil Red O staining for lipid accumulation assessment.

For the curcuminoids intervention study, 24 mice were randomly divided into three groups (n = 8 per group): normal control (NC) group, MASLD model (MM) group, and curcuminoids intervention (CI) group. Curcuminoids were administered daily via oral gavage at a dose of 25 mg/kg body weight, prepared as a stock solution at a concentration of 1.25 mg/mL in 0.5% carboxymethylcellulose sodium (CMC-Na). The gavage volume was 20 mL/kg body weight. All preparations were freshly prepared and protected from light throughout the procedure. During the 12-week experimental period, the NC group received standard chow, the MM group received HFD, and the CI group received HFD supplemented with the curcuminoids via preventive intragastric administration. Daily food and water intake were recorded, and body weight was measured every three days. At the end of the 12-week experiment, serum samples were collected for biochemical assays and circulating exosome isolation. Livers and brown adipose tissues (BAT) were excised, rinsed with cold saline, weighed, and subjected to histopathological examination.

### 2.3 Extraction and characterization of circulating exosomes

#### 2.3.1 Extraction of circulating exosomes

Circulating exosomes were isolated using a qEVoriginal 35 nm size-exclusion chromatography column (IZON, Cat. No. qEVoriginal 35 nm) in combination with a multi-channel microinjection pump (RWD410, Reward Life Sciences, Inc.). A 0.5 mL sample was loaded onto the column, and the pump was activated to maintain pressure equilibrium.

#### 2.3.2 Transmission electron microscopy

After applying 5 μL of purified exosome suspension to a carbon-coated copper grid, the sample was left to adsorb for 10 min at 25 °C. The grid was then stained with 5% phosphotungstic acid (pH 6.8) for 3 min. Excess stain was gently removed with filter paper, and the grid was air-dried for approximately 30 s. The prepared grid was examined using a TEM (JEM-1400, JEOL, Japan) at an accelerating voltage of 80 kV. Representative micrographs were acquired from typical fields.

#### 2.3.3 Western blot analysis

Extraction of exosomal proteins was performed using RIPA buffer supplemented with protease inhibitors, followed by BCA-based quantification. Equal amounts of protein were denatured in loading buffer, separated via SDS-PAGE, and transferred onto PVDF membranes at 180 V for 30 min. After blocking with 5% non-fat milk in Tris-buffered saline containing 0.1% Tween-20 (TBST) for 30 min at room temperature, membranes were incubated overnight at 4 °C with primary antibodies (anti-CD9 1:3,000, anti-CD63 1:2,000, anti-TSG101 1:3,000, anti-calnexin 1:1,000) in 5% BSA/TBST. Following three 10-min TBST washes, HRP-conjugated secondary antibodies (1:3,000) were applied for 30 min at room temperature. Protein bands were visualized using enhanced chemiluminescence and a chemiluminescence detection system.

#### 2.3.4 Nanoparticle tracking analysis

Purified circulating exosomes were diluted 500-700-fold in 1× phosphate-buffered saline and loaded into the sample chamber of a nanoparticle tracking analysis system (Zetaview PMX120-Z, Particle Metrix, Germany). Particle movement was recorded via dynamic light scattering to assess size distribution and concentration of exosomes across groups. From the NTA size distribution data, the mean particle diameter, Span, and Polydispersity Index (PDI) were determined for each sample.

### 2.4 Sample preparation

A total of 100 μL of exosome suspension was aliquoted into a 2 mL centrifuge tube, after which 500 μL of methanol was introduced. The mixture was vortexed for 2 minutes and subjected to ultrasound treatment in an ice bath for 15 minutes. Afterward, the sample was centrifuged at 12,000 × g for 15 minutes at 4 °C. Subsequently, 500 μL of the supernatant was transferred to a new tube and concentrated to near dryness using a vacuum centrifuge. The dried residue was reconstituted in 100 μL of 90% methanol, vortexed thoroughly, and subjected again to ice-bath ultrasonication for 15 minutes. The sample was then centrifuged at 12,000 × g for 15 minutes, and the clarified supernatant was aliquoted into 200 μL sample vials and stored at -20 °C. For quality control (QC), 10 μL from each experimental sample was pooled and aliquoted separately.

### 2.5 UPLC-QTOF-MS/MS

Sample separation was performed using a UPLC M-Class system (Waters, MA, USA) equipped with a Welch Ultimate XB-C18 column (2.1 × 100 mm, 1.8 μm). The chromatographic separation utilized a binary mobile phase system comprising 0.1% formic acid in water (designated as solvent A) and acetonitrile (solvent B). A programed gradient elution profile was executed as follows: starting at 10% B at time zero, increasing linearly to 95% B by 15.0 min, holding at 95% B until 20.0 min, returning to 10% B at 21.0 min, and maintaining this initial condition through 25.0 min. Analytes were introduced via a 2.0 μL injection volume, with the flow rate fixed at 0.3 mL/min and the column temperature regulated at 40 °C.

Mass spectrometric analysis was conducted on a Xevo G2-XS QTOF system equipped with an electrospray ionization source (Waters, MA, USA). Metabolite profiling was primarily conducted in positive mode, while structural elucidation utilized both positive and negative modes. The instrument parameters were as follows: desolvation temperature, 500 °C; capillary voltage, 3.0 kV; cone voltage, 20 V; source temperature, 100 °C; desolvation gas flow, 600 L/h; cone gas flow, 50 L/h; and collision energy, 30-40 eV. Mass spectra were acquired from 50-1200 Da at 1.0 s per scan, using MSE mode for simultaneous collection of precursor and fragment ions.

### 2.6 Data analysis

Raw data acquired from the UPLC-QTOF-MS/MS platform were initially processed using MassLynx v4.1 software (Waters, MA, USA), followed by advanced deconvolution and alignment using MS-DIAL v4. Feature extraction was performed within a retention time window of 1-20 min and a mass range of *m/z* 100–1200 for precursor ions. Fragment ions were analyzed across *m/z* 50-1200, with mass accuracy thresholds of 0.01 Da for MS and 0.015 Da for MS/MS.

In positive ion mode, adducts including [M+H]^+^, [M+Na]^+^, [M+K]^+^, and [M+H–H_2_O]^+^ were considered, while [M-H]^-^, [M+HCOOH-H]^-^, and [M-H_2_O-H]^-^ were used in negative mode for molecular formula prediction. Feature alignment was carried out using a retention time tolerance of 0.1 min and an *m/z* tolerance of 0.02 Da. Peak deconvolution employed a sigma window of 0.6 and a minimum MS/MS intensity threshold of 200. Data normalization was performed using internal standards and the locally weighted scatterplot smoothing (LOWESS) algorithm. To ensure data reliability and consistency, only peaks present in all samples of at least one experimental group were retained for further analysis.

**Figure 1:**
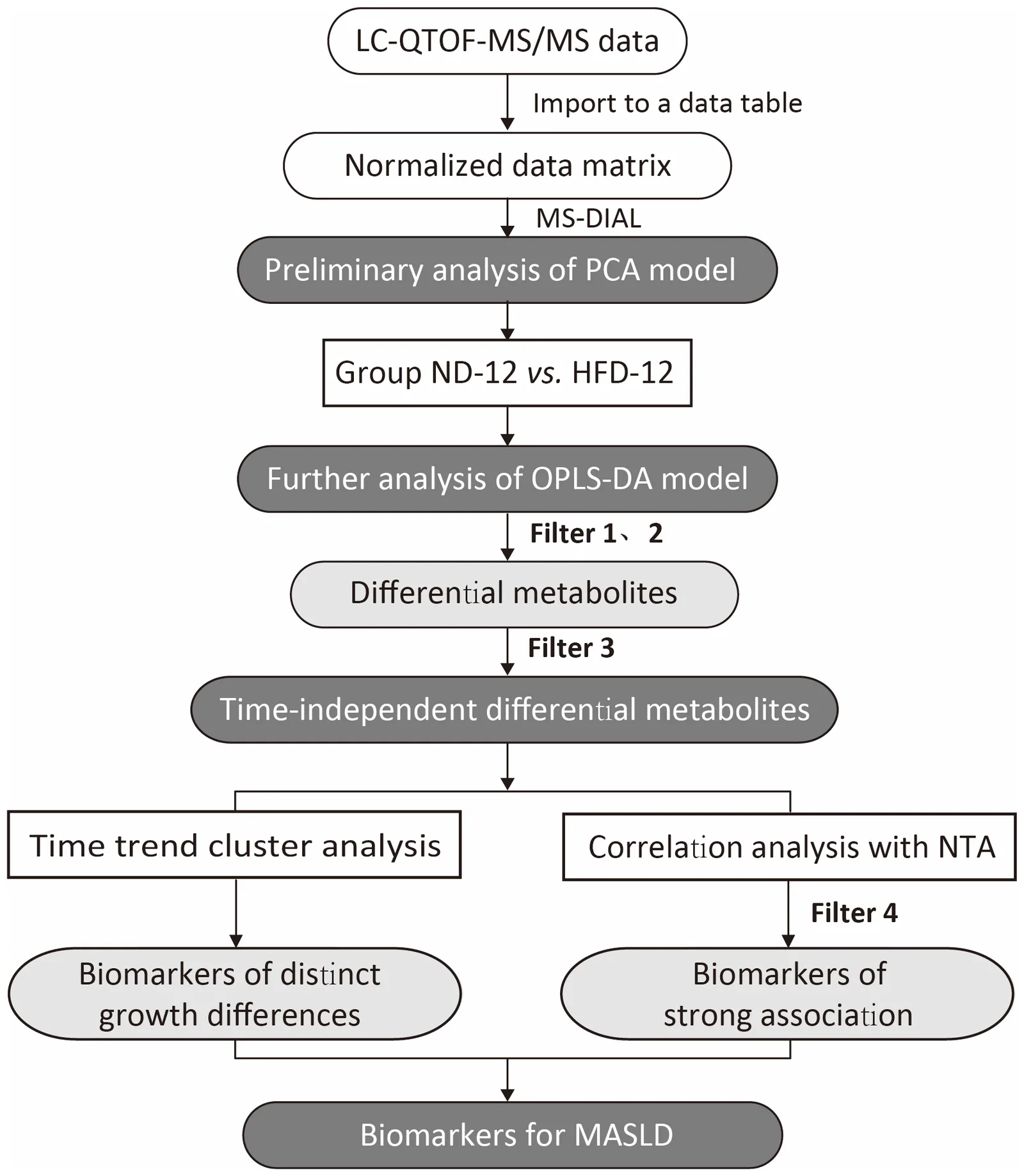
Systematic analysis strategy for biomarker screening in metabolic-associated steatotic liver disease. “ND-12” indicates mice at week 12 of the normal diet. “HFD-12” indicates mice at week 12 of the high-fat diet. PCA, Principal component analysis. OPLS-DA, Orthogonal partial least squares discriminant analysis.

The data processing workflow is illustrated in **Figure 1**. For downstream qualitative analysis, normalized data were filtered using multiple criteria: Filtering Criterion 1: Differential metabolites were retained if they met both of the following conditions: (1) variable importance in projection (VIP) score > 1; and (2) fold change (FC) between the ND-12 and HFD-12 groups > 1.11 or < 0.90, based on multivariate statistical analysis. Filtering Criterion 2: Features were retained if they included at least two adduct ions or were confidently annotated as metabolites by MS-DIAL. Filtering Criterion 3: To eliminate age-dependent metabolic variations, metabolites exhibiting both FC < 0.5 and FC > 1.5 across comparisons of ND-12/ND-9, ND-9/ND-6, ND-6/ND-3, and ND-3/ND-HFD-0 were excluded. Filtering Criterion 4: Metabolites showing significant correlation with circulating exosome particle concentration were retained if they met both of the following conditions: (1) relative pearson correlation coefficient > 0.6; and (2) *p* < 0.05.

For the curcuminoids intervention study, the following filtering criteria were applied: (a) for comparisons between NC vs. MM and MM vs. CI, metabolites were retained if *p* < 0.05 and FC > 1.11 or < 0.90; and (b) *p* < 0.05 and |*r*| > 0.6.

Principal component analysis (PCA) and orthogonal partial least squares discriminant analysis (OPLS-DA) were conducted using SIMCA 14.1 (Umetrics AB, Umeå, Sweden). Data visualization was performed using Origin 2022 (OriginLab Corporation, Northampton, MA, USA). For comparisons between two independent groups, statistical significance was assessed using the non-parametric Mann–Whitney U test (*p* < 0.05). For comparisons involving more than two groups, the Kruskal-Wallis H test was performed, followed by *post hoc* multiple comparisons with Bonferroni correction (*p* < 0.05). All statistical analyses were conducted using SPSS version 27.0 (SPSS Inc., Chicago, IL, USA).

Metabolite annotation was performed using MS-DIAL against its integrated database, which includes the Human Metabolome Database (https://hmdb.ca) among others. Only features with MS/MS matching scores > 80% were retained for further analysis. Quantitative analysis was performed using QuanLynx v4.1 (Waters, Milford, MA, USA). Time-series clustering analysis was conducted using fuzzy c-means clustering with the Mfuzz package in R (v4.2.6). Heatmaps were generated based on Z-score normalized data to visualize time-dependent clustering trends.

## 3 Results

### 3.1 MASLD dynamic model construction

**Figure 2:**
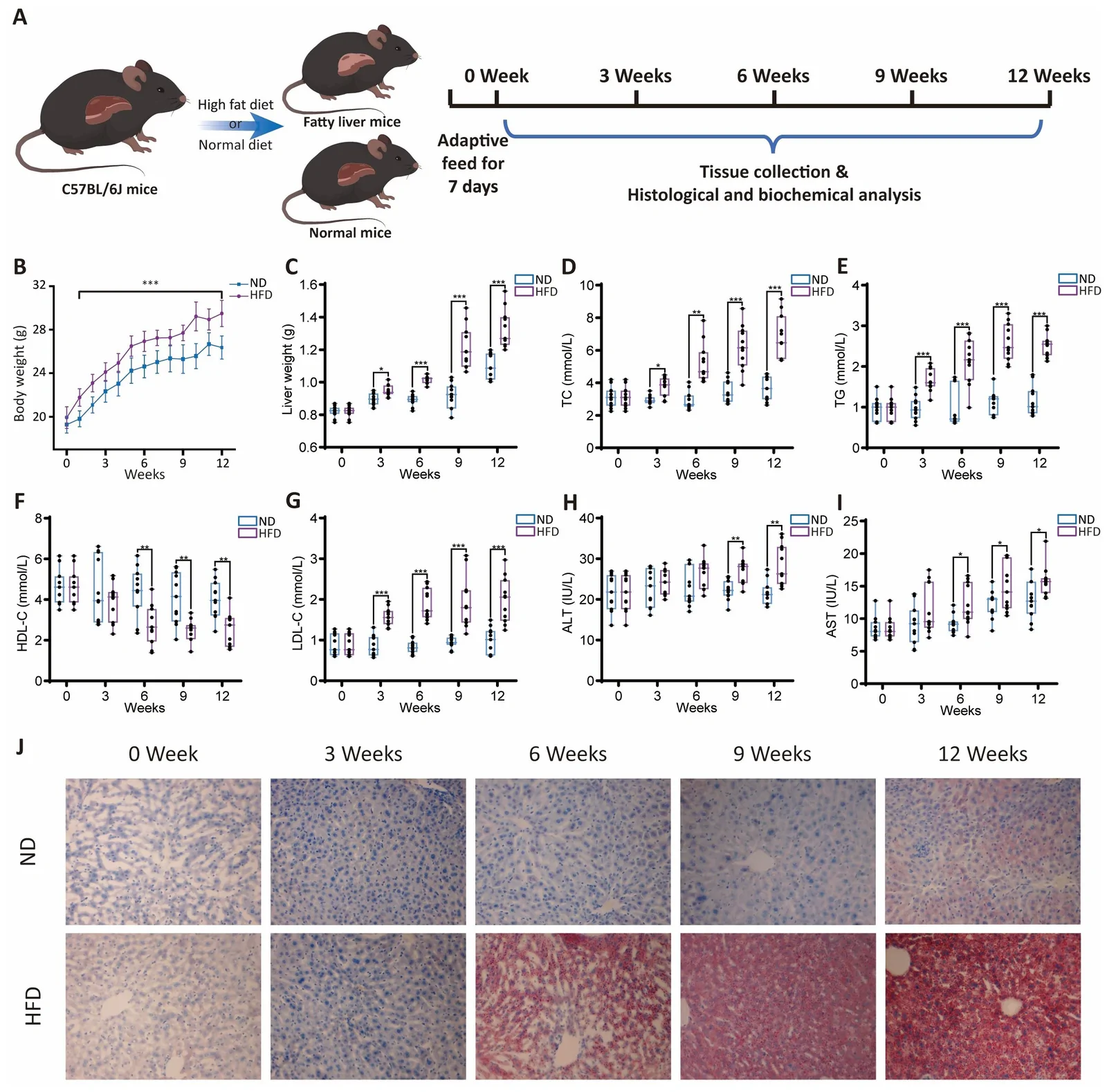
Construction of a dynamic metabolic-associated steatotic liver diseasemouse model through high-fat diet intervention. **(A)** Schematic overview of the MASLD dynamic modeling process. **(B)** Body weight progression of mice over 12 weeks under normal diet (ND) and high-fat diet (HFD) conditions. **(C)** Liver weight changes over time in ND and HFD groups. **(D–I)** Serum lipid and liver function indices including total cholesterol (TC), triglycerides (TG), high-density lipoprotein cholesterol (HDL-C), low-density lipoprotein cholesterol (LDL-C), alanine aminotransferase (ALT), and aspartate aminotransferase (AST) at different time points. **(J)** Representative Oil Red O staining of liver sections from ND and HFD groups at different time points. The box represents the interquartile range, the line within the box indicates the median, and the whiskers extend to the minimum and maximum values (n = 11 mice per group). Statistical significance was determined by the Mann-Whitney U test: *p < 0.05, **p < 0.01, ***p < 0.001 compared with the ND group.

A time-course MASLD model was successfully established using HFD-induced mice over 12 weeks. As shown in **Figure 2A**, the schematic diagram outlines the experimental design for MASLD model construction. Compared to the ND group, HFD-fed mice showed significantly increased body weight starting from week 2 (*p* < 0.05), with progressive differences over time (**Figure 2B**). Liver weight also increased, becoming significantly higher in the HFD group by week 3 (*p* < 0.05) and markedly so by week 12 (*p*< 0.001) (**Figure 2C**). Serum lipid profiles showed time-dependent disruption in HFD mice: TC, TG, and LDL-C levels rose from week 3 and remained elevated (*p*< 0.001), while HDL-C declined significantly at weeks 6, 9, and 12 (*p* < 0.01) (**Figures 2D–G**). Liver enzymes ALT and AST also increased over time, indicating hepatic injury (**Figures 2H, I**). Histologically, hepatic steatosis in HFD mice progressed with time. Mild fat accumulation appeared at week 6, becoming severe by week 12, with notable lipid droplet aggregation and hepatocellular damage (**Figure 2J**).

### 3.2 Characterization of circulating exosomes

**Figure 3:**
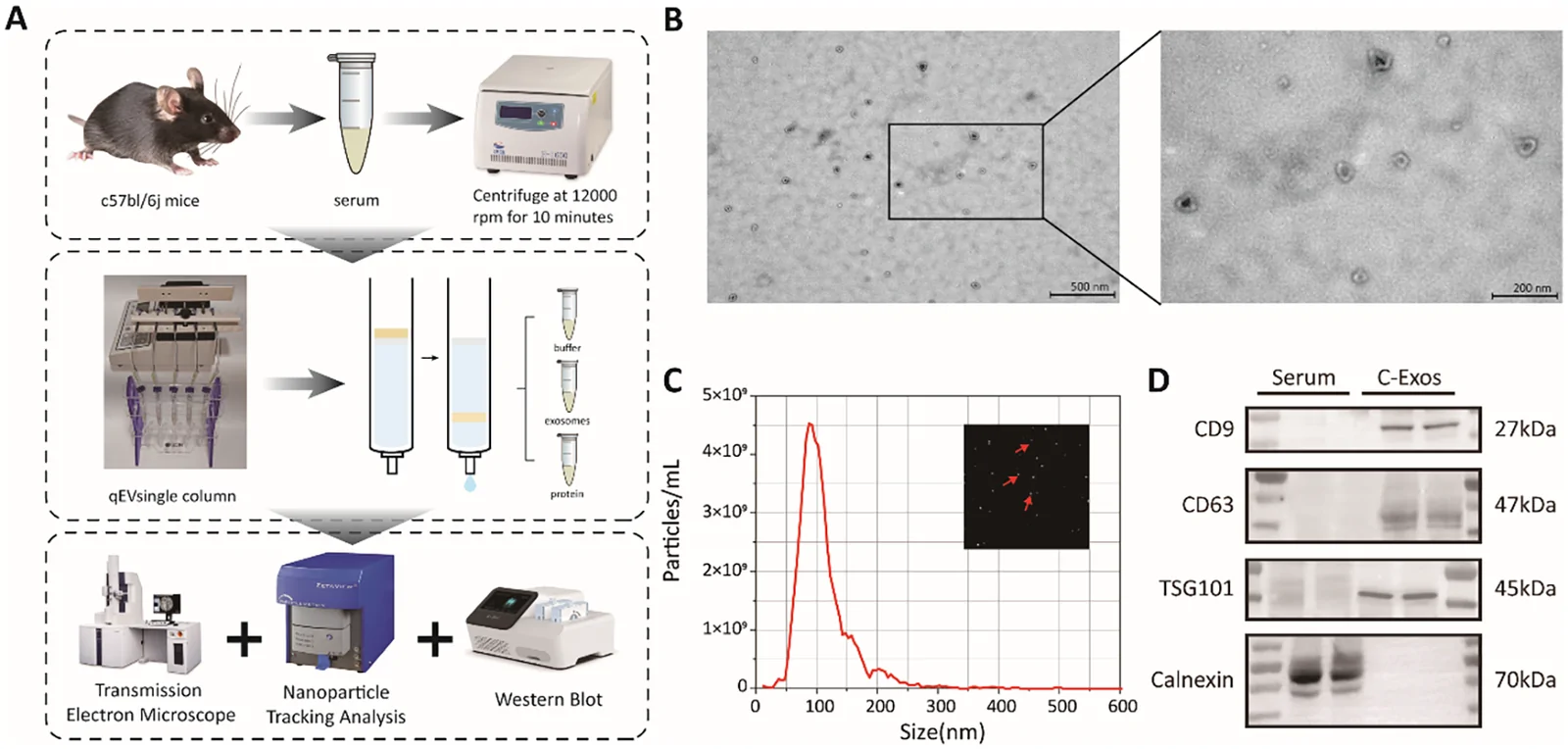
Characterization of circulating exosomes. **(A)** Workflow of circulating exosome isolation and identification. **(B)** Transmission electron microscopy image of exosomes. **(C)** Nanoparticle tracking analysis. **(D)** Western blot analysis of exosomal markers (CD9, CD63, TSG101) and the negative control protein Calnexin. “C-Exos” stands for circulating exosomes.

As shown in **Figure 3A**, the workflow for circulating exosome isolation and identification is illustrated. TEM imaging (**Figure 3B**) revealed typical annular vesicles with well-defined bilayer membrane structures and uniform morphology, without signs of aggregation or contamination. NTA (**Figure 3C**) demonstrated a mean particle diameter of approximately 98 nm, consistent with the expected size range of exosomes (30-150 nm), and a concentration of 4.5 × 10^9^ particles/mL, with a symmetrical size distribution and no impurity peaks. Across all samples, the mean diameters ranged from 96.1 to 127.9 nm (**Supplementary Table 1**), Span values ranged from 0.9 to 1.1 (**Supplementary Table 2**), and PDI values were all below 0.2 (**Supplementary Table 3**), indicating a narrow and highly consistent size distribution across all samples. The size distribution was unimodal in all samples, with a single dominant peak accounting for 100% of detected particles and no secondary peaks, effectively excluding contamination from other non-exosomal particles. Additionally, Western blotting (**Figure 3D**) confirmed the expression of canonical exosomal markers CD9, CD63, and TSG101, whereas the negative marker Calnexin was absent, indicating high sample purity.

### 3.3 Differential metabolites of circulating exosomes associated with MASLD

**Figure 4:**
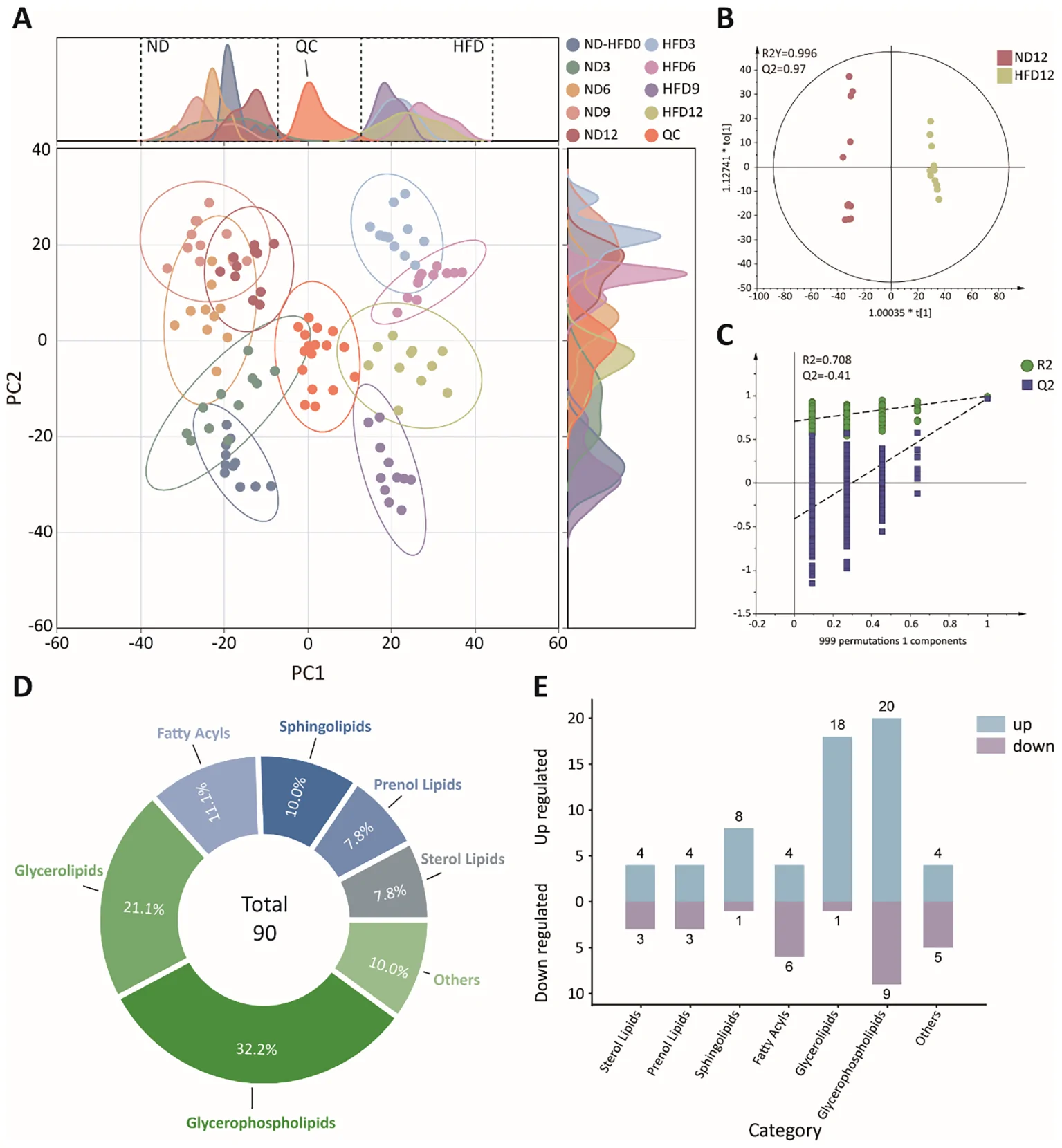
Multivariate analysis and identification of differential metabolites in circulating exosomes. **(A)** Principal component analysis of normal diet, high-fat diet, and quality control (QC) groups. **(B)** Orthogonal Partial Least Squares Discriminant Analysis (OPLS-DA) score plot comparing the Normal Diet 12 weeks (ND-12) and High-Fat Diet 12 weeks (HFD-12) groups. **(C)** Permutation test (999 times) of the OPLS-DA model. **(D)** Classification of differential metabolites between ND-12 and HFD-12 groups. **(E)** Number of upregulated and downregulated metabolites between ND-12 and HFD-12 groups. For PCA and OPLS-DA, n = 11 mice per group. “ND-x” and “HFD-x” indicate mice at week x of the normal diet and high-fat diet, respectively. “ND-HFD0” is the baseline group.

Untargeted metabolomic analysis of circulating exosomes was performed using UPLC-QTOF-MS/MS. PCA revealed distinct separation among the nine groups and QC samples (PC1 = 11.4%, PC2 = 8.1%), with QC samples tightly clustered, indicating stable system performance (**Figure 4A**). Exosomes from ND and baseline samples were distributed on the left side of the PCA plot, while those from HFD groups shifted to the right with a clear time-dependent trend. OPLS-DA was conducted between ND-12 and HFD-12 groups, showing clear discrimination (**Figure 4B**), with R²Y = 0.996 and Q² = 0.97. The 999-time permutation test confirmed model reliability (R² = 0.708, Q² = –0.41; **Figure 4C**).

A total of 3,754 metabolite features were detected from circulating exosome samples using MS-DIAL. After applying filters 1 and 2, 507 differential features were retained for subsequent structural identification. Among them, 90 metabolites were successfully annotated, with approximately 90% classified as lipids, including fatty acids (11.1%), glycerophospholipids (32.2%), glycerolipids (21.1%), sphingolipids (10.0%), prenol lipids (7.8%), sterol lipids (7.8%), and other lipid-like compounds (10.0%) (**Figure 4D**).

Relative to the ND-12 group, the HFD-12 group demonstrated significant bidirectional changes in lipid metabolite profiles. Specifically, 18 glycerolipids and 20 glycerophospholipids were significantly upregulated, with only one and nine compounds downregulated, respectively. In the sterol and prenol lipid classes, four metabolites were increased and three decreased in each subclass. Among sphingolipids, eight were elevated while only one decreased. Fatty acid metabolites displayed mixed trends, with four upregulated and six downregulated species (**Figure 4F**). These results suggest an accumulation of lipid species, particularly glycerol-based lipids, in exosomes from HFD-fed mice. **Supplementary Table 5** provides detailed qualitative data for the identified metabolites, including molecular formulas, exact mass measurements, mass deviations, ion adduct types, fragment ions, retention times, and corresponding identification numbers. **Supplementary Table 6** summarizes the chemical classifications, VIP scores, and FC for the differential metabolites identified between the ND and HFD groups.

### 3.4 Cluster analysis of temporal trends of differential metabolites

To identify candidate biomarkers specifically associated with the pathological progression of MASLD, we first eliminated non-disease-related metabolic fluctuations. Based on the metabolic profiles of mice in the ND-12 group, a third filtering criterion was applied to exclude 19 metabolites linked to developmental stages. Ultimately, 71 differential metabolites were retained, characterized as time-independent MASLD-related features that remained stable under normal dietary conditions but exhibited aberrant changes during disease progression (**Supplementary Table 7** provides the FC ratios between adjacent ND groups). These candidate metabolites were subsequently subjected to heatmap analysis across five time points to explore their dynamic expression profiles throughout the disease course.

**Figure 5:**
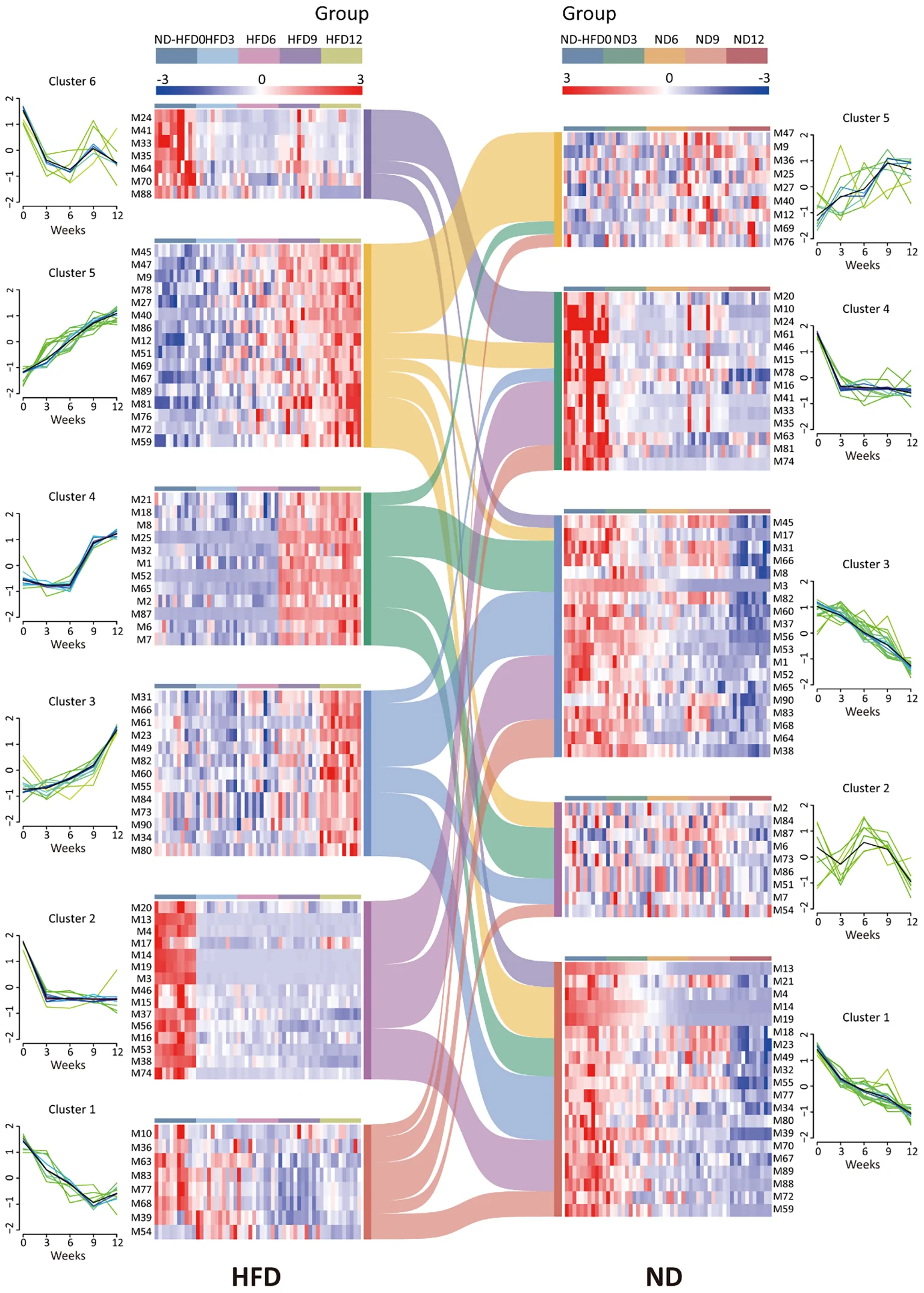
Temporal dynamic pattern analysis of circulating exosomal differential metabolites. “ND-x” and “HFD-x” indicate mice at week x of the normal diet and high-fat diet, respectively. “ND-HFD0” is the baseline group. In the time-series clustering plots, darker line colors represent metabolites whose temporal dynamics more closely align with the cluster model predictions. The heatmap was generated using Z-score normalized intensity data, with the color scale ranging from blue (low intensity) to red (high intensity). In the Sankey diagram, line width reflects the flow intensity of metabolites, and colors distinguish different clusters.

A fuzzy c-means clustering algorithm was employed to classify the temporal expression patterns of these metabolites. As shown in **Figure 5**, six distinct dynamic patterns were identified under HFD conditions: gradual attenuation (Cluster 1), early-phase rapid decline (Cluster 2), progressive accumulation (Clusters 3 and 5), mid-phase surge (Cluster 4), and stable maintenance (Cluster 6). In contrast, five major patterns emerged under the ND: gradual attenuation (Clusters 1 and 3), stable maintenance (Clusters 2 and 5), and early-phase rapid decline (Cluster 4).

The Sankey diagram integrated with temporal clustering analysis revealed the dynamic trajectories and distribution of circulating exosomal metabolites throughout MASLD progression. Comparative analysis indicated that 43 metabolites exhibited distinct temporal transition patterns between the HFD and ND groups. Among these, 29 metabolites in the HFD group displayed sustained accumulation (Clusters 3 and 5), and 12 showed mid-phase surges (Cluster 4). In contrast, the same metabolites in the ND group primarily exhibited gradual attenuation (Clusters 1 and 3), stable levels (Clusters 2 and 5), or early declines (Cluster 4). Notably, these metabolites included 24.4% glycerophospholipids, 36.6% glycerolipids, 7.3% fatty acids, 4.9% sphingolipids, and 9.7% each of prenol lipids, sterol lipids, and other compound classes. Additionally, two metabolites in the HFD group demonstrated a gradual decrease pattern (Cluster 1) that contrasted with a stable trend (Clusters 2 and 5) under normal dietary conditions.

### 3.5 Correlation analysis of particle concentrations of differential metabolites

**Figure 6:**
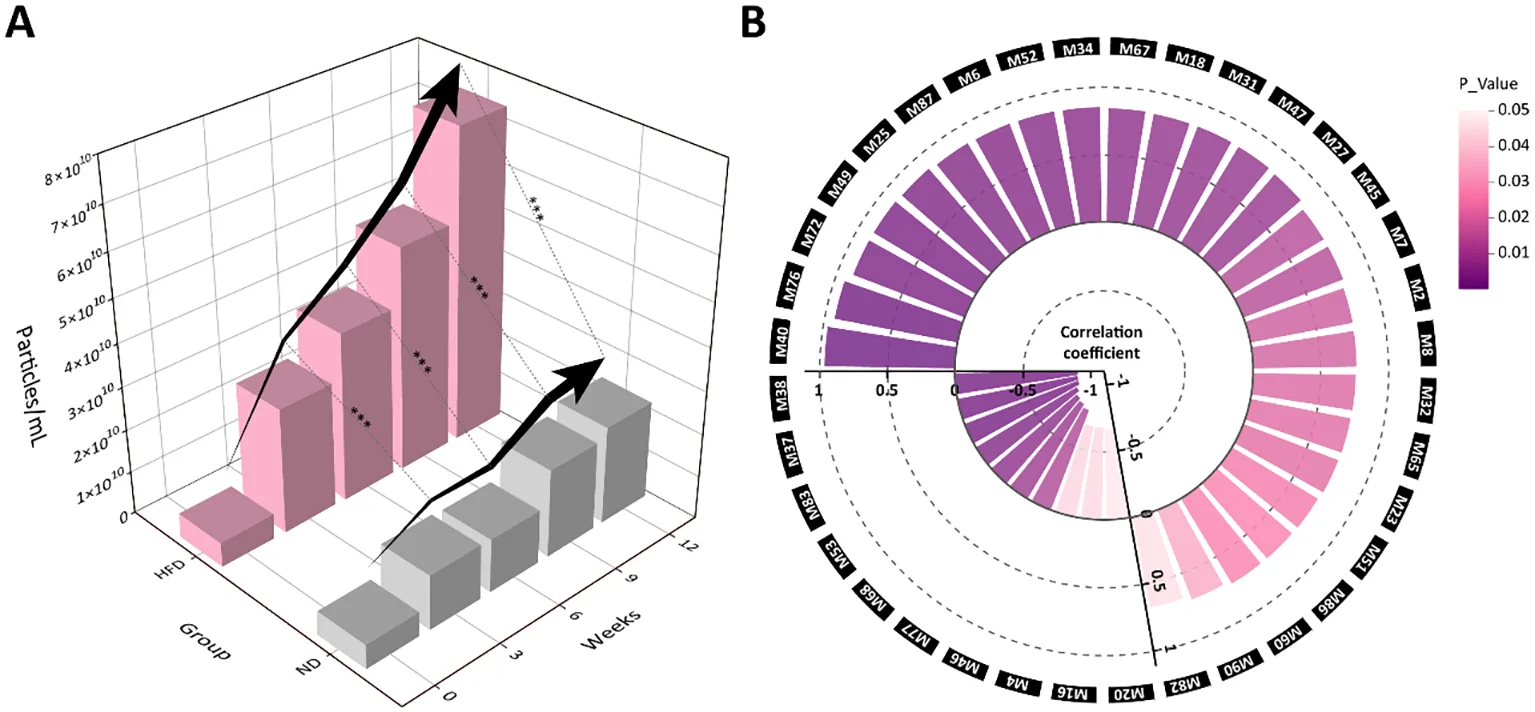
**(A)** The particle concentration of circulating exosomes at different time points. **(B)** Correlation analysis between candidate biomarkers and circulating exosome particle concentration. The depth of color represents the level of statistical significance. “ND” refers to mice fed a normal diet; “HFD” refers to mice fed a high-fat diet. The numbers “0”, “3”, “6”, “9”, and “12” indicate weeks of dietary intervention. Data are presented as mean ± SD (n = 11 mice per group). Statistical significance was determined by the Mann–Whitney U test: ***p < 0.001 compared with the ND group.

NTA was employed to evaluate the particle concentration of circulating exosomes across all experimental groups. As shown in **Figure 6A**, 3D bar plots illustrate a time-dependent increase in exosome concentration in both HFD and ND groups over five time points. Moreover, the extent of this increase varied significantly between the two dietary regimens. In the HFD group, exosome concentrations increased progressively from baseline (week 0) to endpoint (week 12), culminating in a 12.7-fold rise (*p* < 0.001). And the ND group also demonstrated a gradual upward trend, though to a lesser extent, reaching a 4.0-fold elevation (*p* < 0.001).

To investigate metabolic factors associated with exosomal particles, correlation analysis was conducted between exosome concentrations and the 71 time-independent differential metabolites. According to filtering criterion 4 (*p* < 0.05 and |r| > 0.6), 36 metabolites demonstrated strong correlations. As shown in **Figure 6B**, six glycerophospholipids were positively correlated with exosome concentration, while three showed negative correlations. All nine glycerolipids exhibited positive correlations. Among sterol lipids, three metabolites were positively and two negatively correlated. The isoprenoid lipid class included three positively and two negatively correlated metabolites. Within the fatty acid category, one metabolite showed a positive correlation and two showed negative correlations. Additionally, one sphingolipid and three other metabolites were positively correlated, while one metabolite from the “other” class was negatively correlated.

### 3.6 Integrated screening of exosome-associated metabolites relevant to MASLD progression

To further identify MASLD progression-specific biomarkers, an integrated analysis was performed combining time-series clustering of metabolomics data with correlation analysis of exosomal particle concentration. A total of 26 metabolites were co-localized, showing dynamic changes that were significantly synchronized with both MASLD progression and exosome secretion. Detailed information on the trend clustering of biomarkers and their correlations with NTA results is provided in **Supplementary Table 8**. These biomarkers were predominantly composed of glycerolipids and glycerophospholipids, accounting for 57.7% of the total, and included diacylglycerols, monoacylglycerols, lysophosphatidylglycerol, phosphatidic acid, lysophosphatidylcholine, phosphatidylcholine, and phosphatidylserine. Additional classes included prenol lipids, sterol esters, sphingolipids, fatty acids, and other compounds, all exhibiting positive correlations with exosomal concentration. Notably, time-series clustering revealed that these biomarkers showed a consistent upward trend over the course of disease progression in HFD groups, whereas in ND groups, their levels either remained stable or gradually declined over time.

### 3.7 Preventive effects of curcuminoids on MASLD

**Figure 7:**
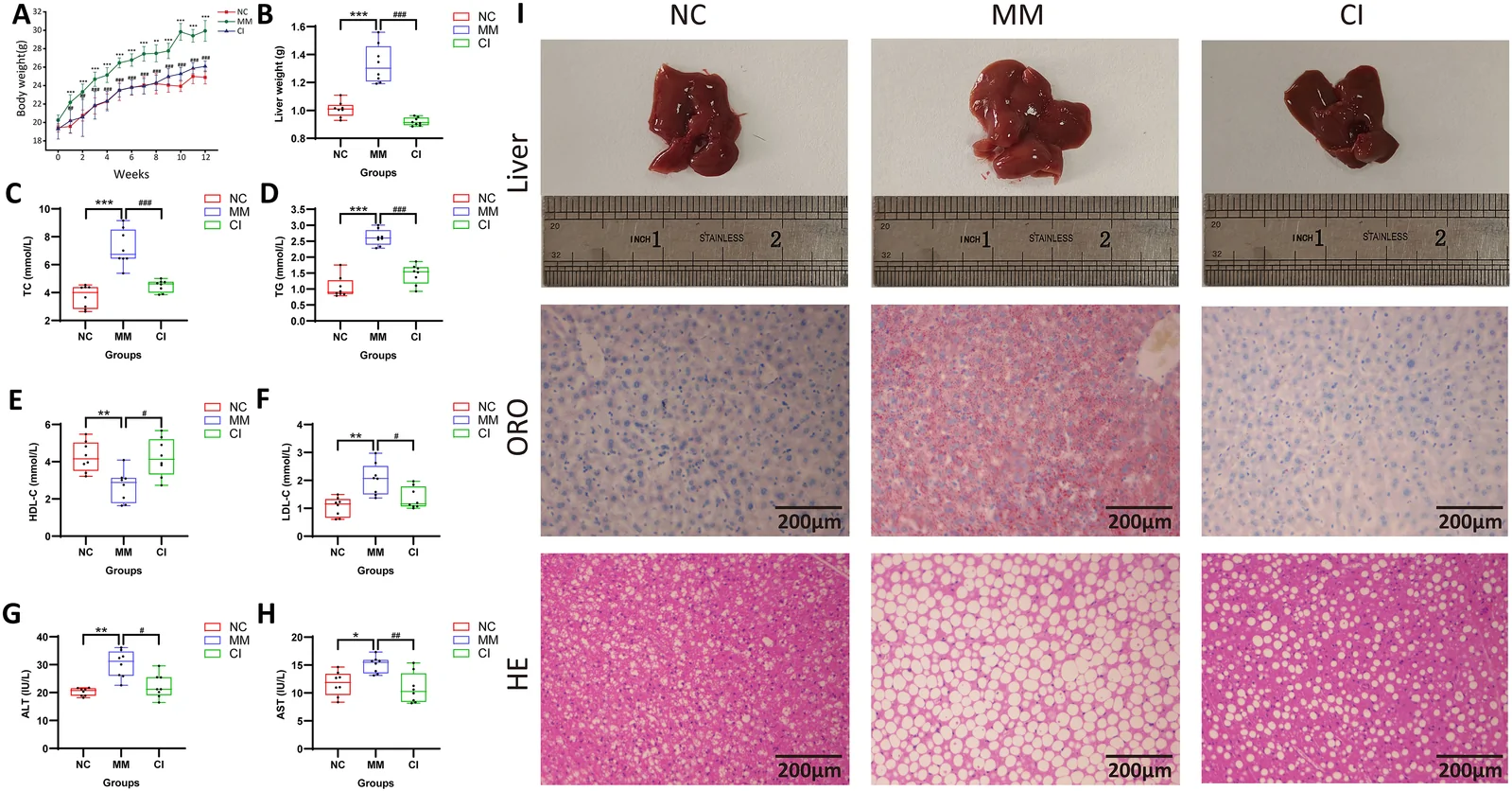
Preventive effects of the curcuminoids on HFD-induced MASLD. **(A)** Body weight gain over the 12-week experimental period in NC, MM, and CI groups. **(B)** Liver weight at week 12 in NC, MM, and CI groups. **(C)** Serum total cholesterol (TC) levels in NC, MM, and CI groups. **(D)** Serum triglycerides (TG) levels in NC, MM, and CI groups. **(E)** Serum low-density lipoprotein cholesterol (LDL-C) levels in NC, MM, and CI groups. **(F)** Serum high-density lipoprotein cholesterol (HDL-C) levels in NC, MM, and CI groups. **(G)** Serum alanine aminotransferase (ALT) levels in NC, MM, and CI groups. **(H)** Serum aspartate aminotransferase (AST) levels in NC, MM, and CI groups. **(I)** Representative images of gross liver appearance, Oil Red O staining (OR) of liver sections (scale bar = 200 μm), and H&E staining (HE) of brown adipose tissue (BAT) sections (scale bar = 200 μm) from NC, MM, and CI groups. NC, normal control; MM, MASLD model; CI, curcuminoids intervention. The box represents the interquartile range, the line within the box indicates the median, and the whiskers extend to the minimum and maximum values (n = 8 mice per group). Statistical significance was determined by the Kruskal-Wallis H test. *p < 0.05, **p < 0.01, ***p < 0.001 vs. NC group; ^#^*p* < 0.05, ^##^*p* < 0.01, ^###^*p* < 0.001 vs. MM group.

As shown in **Figures 7A, B**, HFD feeding significantly increased body weight gain and liver weight in the MM group compared with the NC group (*p* < 0.001), whereas these parameters were markedly reduced in the CI group (*p* < 0.001). These results indicate that the curcuminoids effectively alleviates HFD-induced weight gain and hepatic enlargement. Serum TC, TG, and LDL-C levels were significantly elevated in the MM group compared with the NC group, while HDL-C levels were significantly decreased. Notably, these abnormal changes were significantly reversed in the CI group (MM vs. NC, *p* < 0.01; CI vs. MM, *p* < 0.05; **Figures 7C–F**). Similarly, serum ALT and AST levels were significantly elevated in the MM group and were significantly reduced in the CI group (*p* < 0.01; **Figures 7G, H**).

**Figure 7I** illustrates the histopathological findings. Gross liver appearance: The NC group showed dark red livers with smooth surfaces and normal size and morphology. The MM group exhibited markedly enlarged livers with pale coloration, indicative of HFD-induced fat deposition and hepatic parenchymal damage. The CI group showed improved liver appearance, with size and color approaching those of the NC group. Oil Red O staining: The NC group showed only scattered small orange-red lipid droplets in hepatocytes. The MM group displayed extensive diffuse orange-red lipid droplet accumulation, indicating significant hepatic steatosis. The CI group showed reduced lipid droplet deposition compared with the MM group. H&E staining of brown adipose tissue: The NC group exhibited typical multilocular adipocyte structures with uniformly distributed lipid droplets. The MM group showed markedly enlarged adipocytes with disrupted multilocular structures, displaying unilocular vacuolar changes. The CI group showed a reduced proportion of vacuolated cells, with cell morphology and lipid droplet distribution approaching those of the NC group.

### 3.8 Targeted metabolomic analysis of circulating exosomal biomarkers

**Figure 8:**
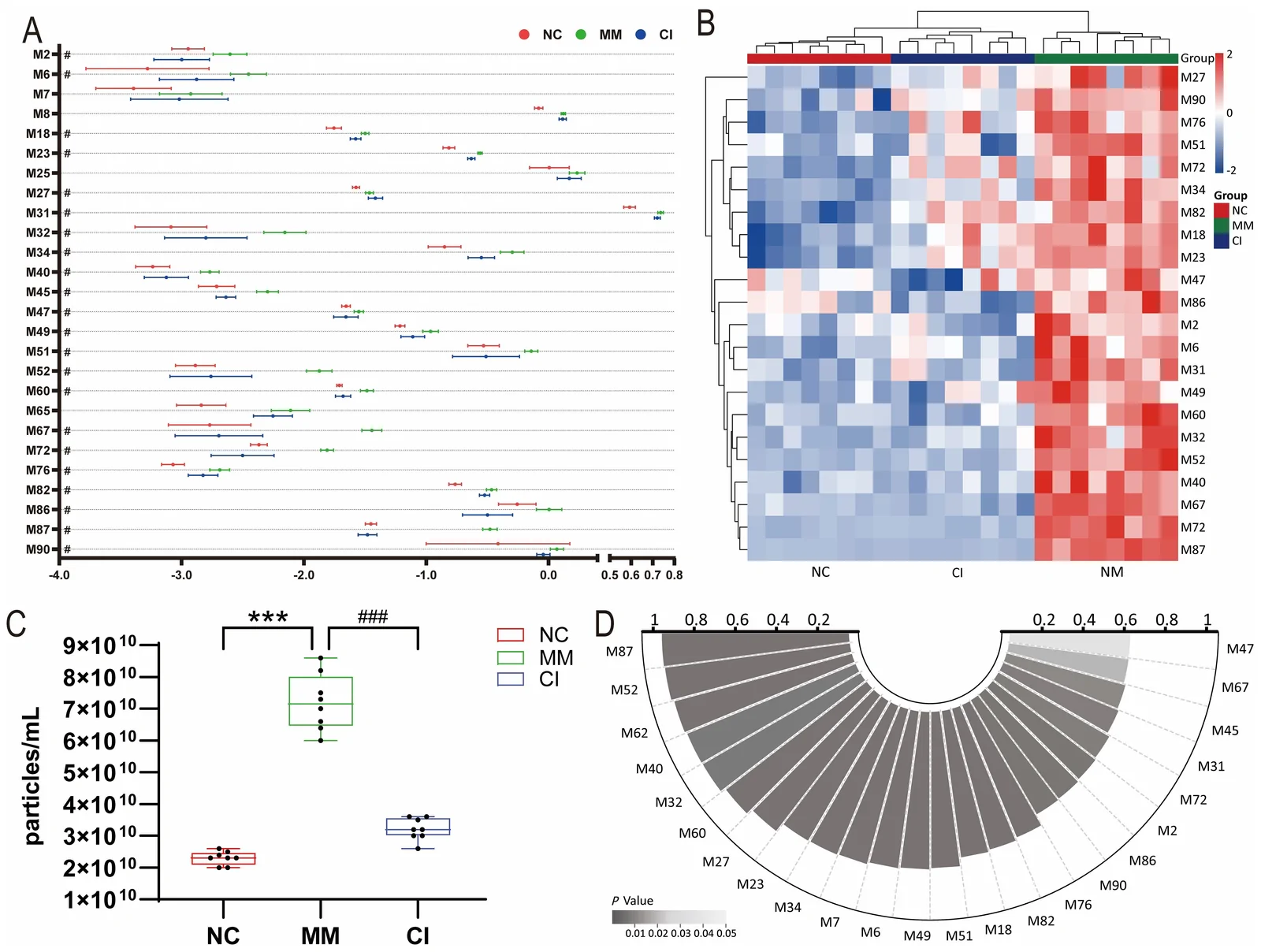
**(A)** Relative abundances of the 26 circulating exosomal biomarkers in the NC, MM, and CI groups. Data are shown as mean ± SD after log10 transformation. # indicates reversal of biomarker levels in the CI group relative to the MM group. **(B)** Hierarchical clustering heatmap of the 22 retained biomarkers across NC, MM, and CI groups. Red indicates upregulation, blue indicates downregulation. **(C)** Exosomal particle concentration in NC, MM, and CI groups determined by NTA. **(D)** Correlation analysis between the 22 biomarkers and exosomal particle concentration. All 22 biomarkers exhibited significant positive correlations (|*r*| > 0.6, *p* < 0.05). NC, normal control; MM, MASLD model; CI, curcuminoids intervention. The box represents the interquartile range, the line within the box indicates the median, and the whiskers extend to the minimum and maximum values (n = 8 mice per group). Statistical significance was determined by the Kruskal-Wallis H test. ***p < 0.001 vs. NC group; ^###^*p* < 0.001 vs. MM group.

To quantify the changes in the 26 identified biomarkers following CF intervention, targeted metabolomic analysis was performed on circulating exosome samples from the NC, MM, and CI groups. The relative abundances of these biomarkers were compared across the three groups (**Figure 8A**). All biomarkers were elevated in the MM group compared with the NC group, whereas CF administration reduced the levels of the majority of these biomarkers, indicating a reversal in the CI group relative to the MM group.

Then the 26 identified biomarkers previously identified were subjected to statistical filtering according to criterion (a). Twenty-two biomarkers were retained for further evaluation of the CI effects on circulating exosomal metabolic regulation during MASLD progression. Hierarchical clustering heatmap based on Z-score normalization showed that, compared with the NC group, the relative abundances of all 22 biomarkers were significantly upregulated in the MM group, suggesting HFD-induced lipid peroxidation, inflammatory responses, and membrane lipid metabolic disturbances (**Figure 8B**). The metabolite abundances in the CI group were significantly reduced compared with the MM group, with ten biomarkers returning to NC group levels (*p* > 0.05 or 0.90 < FC < 1.11). These results indicate that curcuminoids significantly downregulates circulating exosomal biomarkers associated with MASLD progression and partially restores protective metabolite homeostasis.

### 3.9 Correlation analysis of biomarkers with exosomal particle concentration

NTA was performed to evaluate exosomal particle concentrations across the three groups. As shown in **Figure 8C**, exosomal particle concentration in the MM group was significantly higher than that in the NC group (*p* < 0.001), consistent with previous findings that HFD promotes exosome release from hepatocytes. Furthermore, exosomal particle concentration in the CI group was significantly lower than that in the MM group (*p* < 0.001), though still higher than NC group levels, suggesting that the curcuminoids partially reverses metabolic stress-induced exosome hypersecretion, potentially through regulation of lipid raft-dependent secretory pathways.

Correlation analysis was performed between the candidate biomarkers and exosomal particle concentrations. As shown in **Figure 8D**, under screening criterion (b), 24 metabolites exhibited significant positive correlations with exosomal particle concentration. In the CI group, both biomarker levels and exosomal particle concentrations decreased in parallel. Integration of quantitative analysis with correlation analysis further validated the regulatory effects of the curcuminoids on MASLD. As summarized in **Supplementary Table 9**, compared with the MM group, 22 MASLD-associated biomarkers exhibited significant downward trends in the CI group, encompassing key metabolite classes including glycerolipids, glycerophospholipids, sterol lipids, and prenol lipids. Glycerolipids and glycerophospholipids together accounted for 68% of the metabolite profile, including DG, MG, lyso-PG, PA, lyso-PC, PC, and PS. These findings establish a quantitative association between circulating exosome dynamics and lipid metabolite concentrations, suggesting that exosome-mediated lipid signaling may play a role in the metabolic effects of the curcuminoids.

## 4 Discussion

A dynamic mouse model of MASLD progression was successfully established through high-fat diet feeding. By integrating untargeted metabolomics with temporal clustering analysis and correlation analysis of exosomal particle concentrations, the dynamic metabolic alterations in circulating exosomes were systematically characterized across multiple time points. This approach led to the identification of 26 predominantly lipid metabolites as reliable candidate biomarkers, which exhibited a high degree of synchrony with both MASLD progression and exosome secretion dynamics. Unlike conventional cross-sectional biomarker studies, the present study adopted a longitudinal design coupled with filtering criteria, enabling the identification of biomarkers that are both temporally informative and functionally linked to exosome dynamics. Subsequently, the curcuminoids administration downregulated 22 of these biomarkers and reduced exosome secretion, supporting the pharmacological relevance of the identified exosomal lipid signatures.

Metabolomic profiling revealed 71 differentially abundant metabolites in circulating exosomes associated with MASLD, predominantly comprising glycerolipids, glycerophospholipids, fatty acids, and sphingolipids. This specific enrichment pattern aligns closely with the well-established pathological drivers of MASLD, including lipid metabolic dysregulation, inflammation, and insulin resistance (11, 26, 27). Previous studies have elucidated the functional roles of these exosomal lipids in disease progression. Glycerolipids can exacerbate hepatic lipid accumulation by interfering with lipid droplet metabolism and mitochondrial β-oxidation (28). Similarly, sphingolipid metabolites such as sphingosine have been shown to induce peripheral insulin resistance through inhibition of AKT phosphorylation, while also activating the NLRP3 inflammasome in Kupffer cells to promote a pro-inflammatory microenvironment (29, 30). Beyond these metabolic and inflammatory effects, exosome-associated lipids may also contribute to fibrogenesis: palmitic acid carried by exosomes can induce endoplasmic reticulum stress in liver sinusoidal endothelial cells, thereby promoting liver fibrosis (31). Furthermore, cholesterol derivatives can amplify lipotoxic signaling by modulating membrane lipid raft integrity (32). Collectively, circulating exosomes may act as molecular vectors of lipid signals, actively participating in the modulation of the hepatic metabolic microenvironment during MASLD.

To further capture the dynamic changes of metabolite signals in circulating exosomes during disease progression, temporal clustering analysis was performed. The results revealed distinct temporal patterns of differential metabolites between MASLD mice and controls. Glycerolipids and glycerophospholipids were the most abundant classes, highlighting a progressive and continuous lipid metabolic imbalance throughout MASLD development. This dynamic view is corroborated by existing literature: lysophospholipids and phosphatidylcholines, key components of oxidized LDL, are known to induce hepatocyte apoptosis via the ER stress-JNK-CHOP/PUMA signaling axis (33, 34). Concurrently, the accumulation of glycerolipids can further disrupt insulin signaling and mitochondrial function by exacerbating insulin resistance and oxidative stress (35). Thus, these temporal patterns suggest that metabolic reprogramming during MASLD progression occurs in a coordinated and stage-dependent manner.

Additionally, to determine whether changes in differential metabolites were functionally related to exosome secretion, nanoparticle tracking analysis and correlation analysis were employed, revealing 36 metabolites whose abundances correlated significantly with exosomal particle concentration. These metabolites were again predominantly glycerolipids, glycerophospholipids, and sphingolipids. The enhanced exosome secretion observed is likely a consequence of the lipid dysregulation and cellular stress inherent to MASLD progression (36). The positive correlation of glycerolipids with exosome concentration suggests that exosomes may actively transport these lipids, facilitating paracrine lipotoxicity and lipid accumulation in recipient hepatocytes (37). The positive correlation of glycerophospholipids implies their potential role in modulating inflammatory pathways, such as NF-κB and JNK, in recipient cells (38). Similarly, the correlation of sphingolipids with increased exosome secretion may reflect their involvement in activating Kupffer cell NLRP3 inflammasomes, thereby driving fibrosis (39). Interestingly, a negative correlation was observed for certain glycerolipids. This inverse relationship may point to a competitive regulatory mechanism between lipid droplet formation and exosome biogenesis. In early MASLD, excessive lipid droplet accumulation could suppress the packaging of certain lipids into exosomes, whereas in later stages, a compensatory increase in exosome secretion might serve to alleviate cellular stress by exporting toxic lipids (40, 41). This dynamic shift in lipid partitioning between intracellular storage and exosomal export may represent a key mechanistic link in the transition from simple steatosis to steatohepatitis and fibrosis—a hypothesis that warrants further investigation through targeted functional experiments.

Based on an integrative analysis incorporating both temporal dynamics and secretory associations, 26 exosome-derived metabolites were ultimately identified as robust candidate biomarkers for MASLD progression. These metabolites were characterized by significant temporal evolution throughout the disease course and high synchrony with exosome secretion dynamics. Among these, phosphatidylcholines positively correlated with exosome abundance exhibited increasing relative levels over time in HFD-fed mice, potentially reflecting a remodeling of exosomal lipid composition from phosphatidylethanolamine-dominant to phosphatidylcholine-dominant profiles in intestinal epithelial cells. Mechanistically, phosphatidylcholine may activate the aryl hydrocarbon receptor, thereby suppressing key insulin signaling molecules including IRS-2, PI3K, and Akt, ultimately contributing to insulin resistance (42). Secondly, the accumulation of phosphatidylglycerol in adipocyte-derived exosomes may promote lipid droplet formation and exacerbate hepatic lipid overload (43). Thirdly, lysophosphatidylcholine may participate in disease progression through dual mechanisms: promoting exosome release via activation of the DR5-dependent apoptotic signaling pathway, and, once elevated on exosomal membranes, triggering a cascade of exosome release from neighboring cells to amplify inflammatory responses (44). Fourthly, hepatocyte-derived exosomes enriched in phosphatidylserine may bind to surface receptors on macrophages, inducing pro-inflammatory cytokine production and driving hepatic inflammation and fibrosis (45). Therefore, the dynamic alterations in exosomal lipid composition not only mirror the metabolic dysregulation characteristic of MASLD but also actively participate in disease progression through lipid-mediated signaling—a finding enabled by a dual-criterion filtering strategy integrating temporal evolution and secretion synchrony.

The intervention study with curcuminoids provided functional validation of the candidate biomarkers. The curcuminoids administration significantly improved serum lipid profiles, reduced liver enzyme levels, and ameliorated hepatic steatosis and brown adipose tissue injury, consistent with the known metabolic benefits of the curcuminoids (46, 47). Notably, 22 of the 26 biomarkers were significantly downregulated by the curcuminoids, with 10 returning to levels comparable to those of the NC group. These changes were accompanied by a concurrent reduction in exosomal particle concentration, and the 22 biomarkers retained their positive correlations with exosome concentration following intervention. This consistency between biomarker modulation and exosome secretion dynamics suggests that the metabolic effects of the curcuminoids may be mediated, at least in part, through pathways that converge on exosome biogenesis or release. Previous studies have shown that curcuminoids improve lipid metabolism by modulating key transcriptional regulators involved in lipogenesis and cholesterol synthesis (48, 49). The precise molecular targets of the curcuminoids remain to be elucidated. Nevertheless, the present findings suggest that the curcuminoids may modulate exosome-associated lipid metabolites, including glycerolipids and cholesterol derivatives known to contribute to cellular stress upon accumulation. Future studies employing exosome inhibitors would be required to determine whether the effects of curcuminoids on MASLD progression are indeed mediated through exosome-associated lipid pathways.

Several limitations of this study should be acknowledged. Although the high-fat diet-induced mouse model recapitulates key metabolic features of human MASLD, inherent interspecies differences limit direct translatability, necessitating validation in human cohorts. The use of untargeted metabolomics provides broad coverage but precludes absolute quantification. In addition, circulating exosomes represent a mixed population derived from multiple tissues, including liver, brown adipose tissue, and intestine; while the observed lipid signatures may reflect contributions from various sources, the present study cannot definitively assign specific lipid species to their tissue of origin. Future studies employing tissue-specific EV surface markers, such as ASGPR1^+^ for hepatocyte-derived exosomes or CD36^+^ for adipocyte-derived exosomes, will be necessary to trace biomarker provenance and further validate the clinical utility of these candidate lipids. Moreover, BAT injury was observed in HFD-fed mice, but given that the present study employed bulk circulating exosome profiling without tissue-specific origin tracing, a direct link between BAT dysfunction and the observed exosomal lipid signatures cannot be established. Whether BAT-derived exosomes contribute to the circulating lipid alterations reported here remains to be determined in future studies employing tissue-specific exosome labeling or surface marker-based sorting approaches. The present study employed a preventive dosing regimen, with curcuminoids co-administered with HFD from baseline. While this design robustly demonstrates the prophylactic metabolic benefits of curcuminoids, whether the exosome-normalizing effects observed here would persist in established disease remains an open question. Future studies with a therapeutic reversal design, in which curcuminoid administration is initiated after histological steatosis is confirmed (e.g., at week 6 of HFD feeding), will provide a more clinically relevant assessment of the intervention’s translational potential. Furthermore, the present study did not benchmark the identified exosomal lipids against non-exosomal circulating fractions. Future studies comparing exosome-enriched and exosome-depleted fractions would help establish exosome-specific signal enrichment and further validate their biomarker potential.

Despite these limitations, this study provides a comprehensive temporal landscape of circulating exosomal lipid alterations during MASLD progression and establishes a dynamic framework for early diagnosis and real-time monitoring. The dual-criteria strategy integrating temporal dynamics with secretion coupling represents a methodological advance that may inform future biomarker discovery efforts across other disease contexts. Overall, these findings position circulating exosomal lipids as promising non-invasive candidates for early MASLD detection and longitudinal monitoring. Furthermore, the demonstration that the curcuminoids modulates the dynamic trajectory of exosomal lipids adds a pharmacological dimension to the biomarker discovery, suggesting that the identified panel may also serve as a readout for therapeutic efficacy. Overall, these findings position circulating exosomal lipids as promising non-invasive candidates for early MASLD detection and longitudinal monitoring, while also offering experimental evidence for the metabolic effects of the curcuminoids in MASLD.

## 5 Conclusion

By integrating untargeted metabolomics with nanoparticle tracking analysis, this study characterized the dynamic trajectory of circulating exosomal lipids in a mouse model of MASLD progression and evaluated the modulatory effects of the curcuminoids on the identified biomarker panel. Twenty-six lipid biomarkers were identified, distinguished by significant temporal trajectories and strong synchrony with exosome secretion dynamics. The dynamic changes in exosomal lipid composition mirrored MASLD-associated metabolic dysregulation and may play a role in disease progression, potentially through lipid-mediated signaling. In the intervention study, curcuminoids downregulated 22 of these biomarkers and reduced exosome secretion, suggesting a potential association with the identified lipid signatures. This study offers a potential framework for early diagnosis and monitoring, while also providing preliminary experimental evidence for the metabolic effects of the curcuminoids in MASLD. However, further validation in human cohorts is required to confirm the translational relevance of these findings.

## Data Availability

The raw data supporting the conclusions of this article will be made available by the authors, without undue reservation.
