# Supplementary material for: The dynamic trajectory of circulating exosome components in high-fat diet-induced metabolic-associated steatotic liver disease and its modulation by curcuminoids
Source: Front Endocrinol (Lausanne). 2026 Sep 11;17:1933356. doi: 10.3389/fendo.2026.1933356 (PMC13612309; doi:10.3389/fendo.2026.1933356)
Supplement: Supplementary file 1 [file Table1.docx]

**Supplementary Contents**

**Supplementary Tables**

**Table S1.** Mean particle diameter (nm) of circulating exosomes in individual mice from the time‑course cohort across normal diet (ND) and high‑fat diet (HFD) groups at weeks 0, 3, 6, 9, and 12.

| **Sample ID** | **ND-0** | **ND-3** | **ND-6** | **ND-9** | **ND-12** | **HFD-3** | **HFD-6** | **HFD-9** | **HFD-12** |
| --- | --- | --- | --- | --- | --- | --- | --- | --- | --- |
| 1 | 108.8 | 108.9 | 109.5 | 117.1 | 113.5 | 106.2 | 110.0 | 127.9 | 110.7 |
| 2 | 112.1 | 104.3 | 107.3 | 112.4 | 114.8 | 106.4 | 111.3 | 106.3 | 104.8 |
| 3 | 117.4 | 126.8 | 118.9 | 111.2 | 106.5 | 111.1 | 110.3 | 107.4 | 106.7 |
| 4 | 113.5 | 113.5 | 112.2 | 108.6 | 111.8 | 104.9 | 118.1 | 117.6 | 105.6 |
| 5 | 108.7 | 127.0 | 112.2 | 126.2 | 117.0 | 112.3 | 116.6 | 110.4 | 125.9 |
| 6 | 117.5 | 118.6 | 127.7 | 109.0 | 117.8 | 107.9 | 116.9 | 112.4 | 108.1 |
| 7 | 116.3 | 114.9 | 119.8 | 118.9 | 106.2 | 106.5 | 114.7 | 112.7 | 111.8 |
| 8 | 110.0 | 104.0 | 111.8 | 114.5 | 114.2 | 119.5 | 112.2 | 125.1 | 111.4 |
| 9 | 106.2 | 115.3 | 123.6 | 113.5 | 102.9 | 110.5 | 118.9 | 106.6 | 116.5 |
| 10 | 120.0 | 104.0 | 125.2 | 102.3 | 113.2 | 96.1 | 110.0 | 109.9 | 117.9 |
| 11 | 116.1 | 102.0 | 105.3 | 110.0 | 114.7 | 106.9 | 101.3 | 114.5 | 121.6 |

“ND-x” and “HFD-x” indicate mice at week x of the normal diet and high-fat diet, respectively.

**Table S1.** Span values of circulating exosomes in individual mice from the time‑course cohort across normal diet (ND) and high‑fat diet (HFD) groups at weeks 0, 3, 6, 9, and 12.

| **Sample ID** | **ND-0** | **ND-3** | **ND-6** | **ND-9** | **ND-12** | **HFD-3** | **HFD-6** | **HFD-9** | **HFD-12** |
| --- | --- | --- | --- | --- | --- | --- | --- | --- | --- |
| 1 | 1.0 | 1.0 | 1.0 | 1.1 | 0.9 | 1.0 | 0.9 | 1.0 | 1.0 |
| 2 | 1.1 | 1.0 | 1.1 | 1.0 | 1.0 | 1.1 | 1.0 | 1.0 | 0.9 |
| 3 | 1.1 | 1.1 | 1.0 | 0.9 | 0.9 | 0.9 | 1.1 | 1.0 | 0.9 |
| 4 | 1.1 | 1.0 | 0.9 | 1.1 | 1.1 | 0.9 | 1.1 | 0.9 | 0.9 |
| 5 | 1.1 | 1.1 | 1.0 | 1.1 | 1.1 | 1.0 | 0.9 | 1.0 | 0.9 |
| 6 | 1.1 | 1.1 | 1.0 | 1.0 | 1.1 | 1.0 | 1.1 | 0.9 | 0.9 |
| 7 | 1.1 | 0.9 | 1.0 | 1.0 | 1.0 | 1.0 | 1.0 | 1.0 | 1.0 |
| 8 | 1.0 | 1.0 | 1.1 | 1.1 | 1.1 | 1.0 | 1.0 | 1.0 | 0.9 |
| 9 | 1.1 | 1.1 | 1.0 | 0.9 | 0.9 | 1.0 | 1.0 | 0.9 | 1.1 |
| 10 | 1.1 | 1.0 | 1.1 | 1.0 | 1.0 | 1.0 | 0.9 | 0.9 | 1.0 |
| 11 | 0.9 | 0.9 | 0.9 | 1.0 | 1.1 | 1.0 | 0.9 | 1.0 | 1.0 |

Span = (D90 – D10) / D50, where D10, D50, and D90 represent the particle diameters at the 10th, 50th, and 90th percentiles of the cumulative size distribution, respectively. Span is a measure of size distribution width derived from NTA measurements. All individual Span values ranged from 0.9 to 1.1, with group means consistently below 1.1, indicating a narrow and highly consistent size distribution across all samples and supporting the homogeneity and purity of the exosome preparations. “ND-x” and “HFD-x” indicate mice at week x of the normal diet and high-fat diet, respectively.

**Table S3.** Polydispersity Index (PDI) values of circulating exosomes in individual mice from the time‑course cohort across normal diet (ND) and high‑fat diet (HFD) groups at weeks 0, 3, 6, 9, and 12.

| **Sample ID** | **ND-0** | **ND-3** | **ND-6** | **ND-9** | **ND-12** | **HFD-3** | **HFD-6** | **HFD-9** | **HFD-12** |
| --- | --- | --- | --- | --- | --- | --- | --- | --- | --- |
| 1 | 0.10 | 0.15 | 0.13 | 0.15 | 0.10 | 0.08 | 0.08 | 0.13 | 0.12 |
| 2 | 0.16 | 0.15 | 0.15 | 0.12 | 0.10 | 0.10 | 0.11 | 0.08 | 0.10 |
| 3 | 0.16 | 0.18 | 0.09 | 0.11 | 0.10 | 0.12 | 0.13 | 0.12 | 0.09 |
| 4 | 0.12 | 0.11 | 0.11 | 0.13 | 0.14 | 0.12 | 0.12 | 0.08 | 0.08 |
| 5 | 0.15 | 0.13 | 0.10 | 0.15 | 0.13 | 0.10 | 0.07 | 0.11 | 0.09 |
| 6 | 0.12 | 0.12 | 0.11 | 0.14 | 0.14 | 0.12 | 0.07 | 0.09 | 0.11 |
| 7 | 0.18 | 0.10 | 0.11 | 0.09 | 0.10 | 0.10 | 0.10 | 0.11 | 0.12 |
| 8 | 0.09 | 0.10 | 0.15 | 0.14 | 0.17 | 0.12 | 0.08 | 0.12 | 0.12 |
| 9 | 0.11 | 0.15 | 0.13 | 0.10 | 0.10 | 0.10 | 0.10 | 0.09 | 0.12 |
| 10 | 0.11 | 0.10 | 0.14 | 0.10 | 0.09 | 0.10 | 0.08 | 0.08 | 0.14 |
| 11 | 0.09 | 0.10 | 0.12 | 0.13 | 0.14 | 0.18 | 0.11 | 0.09 | 0.10 |

All individual PDI values were below 0.2, indicating a highly homogeneous particle population with a narrow size distribution across all samples and strongly supporting the high purity of the exosome preparations. “ND-x” and “HFD-x” indicate mice at week x of the normal diet and high-fat diet, respectively.

**Table S4.** Exosome particle concentration (×10¹⁰ particles/mL) in individual mice from the time‑course cohort across normal diet (ND) and high‑fat diet (HFD) groups at weeks 0, 3, 6, 9, and 12.

| **Sample ID** | **ND-0** | **ND-3** | **ND-6** | **ND-9** | **ND-12** | **HFD-3** | **HFD-6** | **HFD-9** | **HFD-12** |
| --- | --- | --- | --- | --- | --- | --- | --- | --- | --- |
| 1 | 0.65 | 1.50 | 0.95 | 1.90 | 2.30 | 3.10 | 4.10 | 5.70 | 7.50 |
| 2 | 0.42 | 1.10 | 1.30 | 2.00 | 2.40 | 2.40 | 3.70 | 4.60 | 6.40 |
| 3 | 0.58 | 1.40 | 1.90 | 2.20 | 2.30 | 3.00 | 3.80 | 5.10 | 7.00 |
| 4 | 0.41 | 1.80 | 1.30 | 1.90 | 2.60 | 3.00 | 3.70 | 4.60 | 6.00 |
| 5 | 0.60 | 1.10 | 1.60 | 2.30 | 2.30 | 3.20 | 3.80 | 5.20 | 7.30 |
| 6 | 0.44 | 0.75 | 0.92 | 2.10 | 2.50 | 2.80 | 3.80 | 4.70 | 6.60 |
| 7 | 0.68 | 0.72 | 1.10 | 1.90 | 2.00 | 2.90 | 4.30 | 5.80 | 8.60 |
| 8 | 0.65 | 1.30 | 0.73 | 2.20 | 2.00 | 2.80 | 4.20 | 5.80 | 8.20 |
| 9 | 0.63 | 1.60 | 1.30 | 2.30 | 2.10 | 3.50 | 3.90 | 5.40 | 7.40 |
| 10 | 0.56 | 1.70 | 1.70 | 1.90 | 2.50 | 3.00 | 3.80 | 4.90 | 6.80 |
| 11 | 0.71 | 1.10 | 0.97 | 1.90 | 2.10 | 2.40 | 4.30 | 5.80 | 8.70 |

“ND-x” and “HFD-x” indicate mice at week x of the normal diet and high-fat diet, respectively.

**Table S5.** Identification information of differential metabolites in UPLC-QTOF-MS/MS analysis.

| **No.** | **RT (min)** | **Tentative identification** | **Formula** | **Mass (m/z)** | **Mass error (ppm)** | **Adduct** | **MS2 fragments** | **MSI level** |
| --- | --- | --- | --- | --- | --- | --- | --- | --- |
| M1 | 7.08 | Macrocarpal B | C_28_H_40_O_6_ | 495.2723 | 6.46 | [M+Na]^+^ | 281.14, 185.05, 183.03, 111.00 | Level 2 |
| M2 | 7.39 | Cucurbitacin D | C_30_H_44_O_7_ | 539.2985 | 15.20 | [M+Na]^+^ | 443.27, 317.22 | Level 2 |
| M3 | 7.46 | 4-Hydroxy-5-(phenyl)-valeric acid-O-glucuronide | C_17_H_22_O_9_ | 393.1162 | 4.07 | [M+Na]^+^ | 161.10, 133.10, 131.09, 129.02 | Level 2 |
| M4 | 7.52 | 3,7,8,15-Scirpenetetrol | C_15_H_22_O_6_ | 321.1314 | 4.36 | [M+Na]^+^ | 267.12, 211.09, 143.07, 141.06 | Level 2 |
| M5 | 7.54 | Citrusin D | C_16_H_22_O_8_ | 365.1212 | -8.49 | [M+Na]^+^ | 283.12, 181.08, 179.08, 165.07 | Level 2 |
| M6 | 7.69 | Cucurbitacin C | C_32_H_48_O_8_ | 583.3247 | 5.83 | [M+Na]^+^ | 385.24, 383.22, 345.24, 343.22 | Level 2 |
| M7 | 8.17 | Tragopogonsaponin A | C_36_H_56_O_10_ | 671.3772 | 8.34 | [M+Na]^+^ | 591.32, 329.15, 185.12, 177.09 | Level 2 |
| M8 | 8.77 | Mevastatin | C_23_H_34_O_5_ | 413.2304 | -4.60 | [M+Na]^+^ | 273.18, 263.20, 249.15, 157.10 | Level 2 |
| M9 | 11.82 | 9,10,13-TriHOME | C_18_H_34_O_5_ | 353.2304 | 2.55 | [M+Na]^+^ | 197.12, 109.10, 101.06, 95.09 | Level 2 |
| M10 | 12.5 | Phytosphingosine | C_18_H_39_NO_3_ | 318.3008 | 0.31 | [M+H]^+^ | 318.30, 300.30, 265.26 | Level 2 |
| M11 | 12.57 | Aminopentol | C_22_H_47_NO_5_ | 406.3532 | -2.21 | [M+H]^+^ | 131.10, 127.11, 120.10, 118.08 | Level 2 |
| M12 | 12.72 | Cyclo(D-trp-D-asp-pro-D-val-leu) | C_31_H_42_N_6_O_7_ | 633.3013 | 1.58 | [M+Na]^+^ | 525.22, 523.26, 438.20, 136.11 | Level 2 |
| M13 | 13.02 | Delmopinol | C_16_H_33_NO_2_ | 272.259 | 0.37 | [M+H]^+^ | 242.21, 130.09, 100.08, 97.10 | Level 2 |
| M14 | 13.4 | N-Palmitoyl Serine | C_19_H_37_NO_4_ | 344.2801 | 4.65 | [M+H]^+^ | 179.18, 125.13, 109.10, 106.04 | Level 2 |
| M15 | 13.55 | 4-O-Methylmelleolide | C_24_H_30_O_6_ | 437.1941 | -2.52 | [M+Na]^+^ | 359.15, 223.07, 221.16, 219.13 | Level 2 |
| M16 | 13.71 | Corticosterone-21-hemisuccinate | C_25_H_34_O_7_ | 469.2203 | -1.92 | [M+Na]^+^ | 287.20, 265.16, 163.11, 161.09 | Level 2 |
| M17 | 13.77 | 9-HPODE | C_18_H_32_O_4_ | 335.2199 | 6.56 | [M+Na]^+^ | 195.14, 135.12, 133.11, 121.10 | Level 2 |
| M18 | 13.86 | DG(16:0) | C_19_H_36_O_5_ | 367.2461 | -7.62 | [M+Na]^+^ | 215.17, 209.15, 185.15, 139.17 | Level 2 |
| M19 | 14.05 | Dodecanoylcarnitine | C_19_H_38_NO_4_ | 344.2801 | -6.39 | [M+H]^+^ | 244.27, 109.10, 95.09 | Level 2 |
| M20 | 14.36 | Dodecanamide | C_12_H_25_NO | 200.2014 | 8.99 | [M+H]^+^ | 95.08, 83.08, 81.06 | Level 2 |
| M21 | 14.89 | MG(12:0) | C_15_H_30_O_4_ | 297.2042 | -2.36 | [M+Na]^+^ | 297.20, 239.20 | Level 2 |
| M22 | 14.93 | 9-HODE | C_18_H_32_O_3_ | 319.225 | 0.63 | [M+Na]^+^ | 195.14, 147.12, 135.12, 133.10 | Level 2 |
| M23 | 14.97 | DG(18:0) | C_21_H_40_O_5_ | 395.2774 | -5.82 | [M+Na]^+^ | 395.28, 251.17, 167.10 | Level 2 |
| M24 | 15.19 | Glycerol tributanoate | C_15_H_26_O_6_ | 325.1628 | -6.46 | [M+Na]^+^ | 320.21, 215.12, 71.05 | Level 2 |
| M25 | 15.28 | Methyl N-({3-[(Propylamino)carbonyl]oxiran-2-Yl}carbonyl)-L-Isoleucyl-L-Prolinate | C_19_H_31_N_3_O_6_ | 398.2291 | 12.81 | [M+H]^+^ | 281.15, 253.16, 241.15, 130.09 | Level 2 |
| M26 | 15.64 | Lyso-PC/16:1 | C_24_H_48_NO_7_P | 516.3067 | -2.32 | [M+Na]^+^ | 184.07, 125.00, 104.11, 86.10 | Level 2 |
| M27 | 15.89 | 16:0/Lyso-PG | C_22_H_45_O_9_P | 507.2699 | -0.99 | [M+Na]^+^ | 255.24 | Level 2 |
| M28 | 15.91 | 20:4/Lyso-PC | C_28_H_50_NO_7_P | 566.3223 | -4.94 | [M+Na]^+^ | 184.07, 125.00, 104.11, 86.10 | Level 2 |
| M29 | 15.94 | Lyso-PE/20:4 | C_25_H_44_NO_7_P | 502.2934 | -5.77 | [M+H]^+^ | 441.29, 361.27 | Level 2 |
| M30 | 15.97 | 18:2/Lyso-PE | C_23_H_44_NO_7_P | 478.2934 | -3.35 | [M+H]^+^ | 337.28, 417.29 | Level 2 |
| M31 | 15.99 | MG(i-14:0) | C_17_H_34_O_4_ | 325.2355 | -3.07 | [M+Na]^+^ | 285.24, 229.22, 211.20 | Level 2 |
| M32 | 16.02 | 24-Methylenecholesterol | C_28_H_46_O | 421.3447 | -6.88 | [M+Na]^+^ | 119.08, 109.10, 93.07 | Level 2 |
| M33 | 16.15 | Glycerol phenylbutyrate | C_33_H_38_O_6_ | 553.2567 | 1.27 | [M+Na]^+^ | 451.21, 439.21, 437.20, 365.18 | Level 2 |
| M34 | 16.27 | PA(33:5-3OH) | C_36_H_61_O_11_P | 701.403 | -0.29 | [M+H]^+^ | 171.00, 152.99 | Level 2 |
| M35 | 16.65 | Cer(d36:3-OH) | C_36_H_67_NO_4_ | 578.5148 | -8.99 | [M+H]^+^ | 578.51, 542.49, 266.29 | Level 2 |
| M36 | 16.67 | Cetylmannoside | C_22_H_44_O_6_ | 427.3036 | -3.98 | [M+Na]^+^ | 345.30, 313.27, 285.28, 165.07 | Level 2 |
| M37 | 16.68 | Melledonol | C_23_H_30_O_8_ | 435.2019 | 8.96 | [M+H]^+^ | 374.18, 338.19, 314.17, 205.10 | Level 2 |
| M38 | 16.8 | 12-[4-(Dimethylamino)-3-hydroxy-6-methyloxan-2-yl]oxy-4-ethyl-5,5',6,11-tetrahydroxy-4'-methoxy-4',5,6',7,9,11,13-heptamethylspiro[3,15,17-trioxabicyclo[12.4.0]octadecane-16,2'-oxane]-2,8-dione | C_37_H_65_NO_14_ | 770.4303 | 4.28 | [M+Na]^+^ | 616.37, 592.37, 574.36, 572.34 | Level 2 |
| M39 | 16.83 | SM(d16:1/5-iso PGF2VI) | C_39_H_73_N_2_O_9_P | 745.5132 | -2.68 | [M+H]^+^ | 767.49, 708.43 | Level 2 |
| M40 | 16.87 | P-18:0/Lyso-PC | C_26_H_54_NO_6_P | 530.3587 | -1.32 | [M+Na]^+^ | 184.07, 125.00, 104.11, 86.10 | Level 2 |
| M41 | 16.93 | Cer(d34:0) | C_34_H_69_NO_3_ | 540.5356 | -4.07 | [M+H]^+^ | 540.53, 284.29 | Level 2 |
| M42 | 17.03 | 18:0/Lyso-PC | C_26_H_54_NO_7_P | 524.3716 | -0.57 | [M+H]^+^ | 184.07, 125.00, 104.11, 86.10 | Level 2 |
| M43 | 17.05 | Lyso-PE/18:0 | C_23_H_48_NO_7_P | 482.3247 | 6.63 | [M+H]^+^ | 341.3 | Level 2 |
| M44 | 17.07 | Lyso-PE/20:3 | C_25_H_46_NO_7_P | 504.309 | 2.78 | [M+H]^+^ | 443.32, 363.29 | Level 2 |
| M45 | 17.15 | MG(17:0) | C_20_H_40_O_4_ | 367.2825 | 0.00 | [M+Na]^+^ | 239.19, 139.11, 125.10, 113.10 | Level 2 |
| M46 | 17.16 | Cholic acid | C_24_H_40_O_5_ | 409.2954 | 8.06 | [M+H]^+^ | 393.26, 359.25, 347.26, 319.22 | Level 2 |
| M47 | 17.25 | N,N-Dimethylsphingosine | C_20_H_41_NO_2_ | 350.3036 | -1.71 | [M+Na]^+^ | 109.10, 95.08, 69.07 | Level 2 |
| M48 | 17.35 | Bufotalin | C_26_H_36_O_6_ | 467.241 | 7.49 | [M+Na]^+^ | 427.25, 373.20, 365.21, 305.14 | Level 2 |
| M49 | 17.36 | Polyglycerol esters of fatty acids | C_24_H_48_O_6_ | 455.3349 | -0.66 | [M+Na]^+^ | 341.30, 267.27, 89.06 | Level 3 |
| M50 | 17.38 | 22:0/Lyso-PE | C_27_H_56_NO_7_P | 538.3873 | -3.90 | [M+H]^+^ | 397.27 | Level 2 |
| M51 | 17.43 | DG(36:3) | C_39_H_70_O_5_ | 619.5302 | -7.10 | [M+H]^+^ | 619.54, 601.51, 393.34 | Level 2 |
| M52 | 17.46 | alpha-Tocopherol acetate | C_31_H_52_O_3_ | 495.3815 | -2.42 | [M+Na]^+^ | 457.36, 359.25, 345.25, 331.22 | Level 2 |
| M53 | 17.48 | Hydroxystrobilurin D | C_26_H_34_O_7_ | 459.2383 | -1.09 | [M+H]^+^ | 411.22, 399.21, 379.19, 369.20 | Level 2 |
| M54 | 17.5 | PE(38:5-OH) | C_43_H_76_NO_9_P | 782.5336 | 6.77 | [M+H]^+^ | 721.50, 641.51 | Level 2 |
| M55 | 17.51 | DG(29:0) | C_32_H_62_O_5_ | 549.4496 | 4.37 | [M+Na]^+^ | 353.31, 341.30, 299.30, 281.28 | Level 2 |
| M56 | 17.6 | Prostaglandin G2 2-glyceryl Ester | C_23_H_38_O_8_ | 443.2645 | 8.35 | [M+H]^+^ | 425.25, 411.28, 353.24 | Level 2 |
| M57 | 17.75 | Digalactosyldiacylglycerol | C_49_H_88_O_15_ | 939.6021 | -10.64 | [M+Na]^+^ | 855.58, 845.52, 931.51, 637.37 | Level 2 |
| M58 | 17.86 | PS(36:1) | C_42_H_80_NO_10_P | 790.5598 | 2.53 | [M+H]^+^ | 685.43, 605.56 | Level 2 |
| M59 | 17.88 | PC(PGF1alpha/16:0) | C_44_H_84_NO_11_P | 834.586 | 6.35 | [M+H]^+^ | 184.07, 125.00, 104.10, 86.10 | Level 2 |
| M60 | 17.96 | Ximenic acid | C_26_H_50_O_2_ | 417.3709 | -3.83 | [M+Na]^+^ | 309.28, 239.20, 211.17, 137.14 | Level 2 |
| M61 | 17.99 | 7a-Hydroxy-cholestene-3-one | C_27_H_44_O_2_ | 423.324 | 1.42 | [M+Na]^+^ | 385.31, 317.25, 273.18, 193.13 | Level 2 |
| M62 | 18.06 | PE-NMe(40:7) | C_46_H_78_NO_8_P | 804.5543 | -9.69 | [M+H]^+^ | 743.54, 663.53 | Level 2 |
| M63 | 18.08 | DG(34:3-OH) | C_37_H_66_O_6_ | 607.4938 | -0.49 | [M+H]^+^ | 559.48, 479.34, 477.36, 463.38 | Level 2 |
| M64 | 18.1 | PC(34:2) | C_42_H_80_NO_8_P | 758.57 | 6.33 | [M+H]^+^ | 184.07, 125.00, 86.10 | Level 2 |
| M65 | 18.34 | Cholylthreonine | C_28_H_47_NO_7_ | 510.3431 | 0.78 | [M+H]^+^ | 347.26, 345.24, 307.22, 305.21 | Level 2 |
| M66 | 18.38 | MG(i-20:0) | C_23_H_46_O_4_ | 409.3294 | -4.40 | [M+Na]^+^ | 369.33, 355.35, 313.32 | Level 2 |
| M67 | 18.46 | PC(36:3) | C_44_H_82_NO_8_P | 806.5676 | 0.00 | [M+Na]^+^ | 184.07, 125.00, 104.11, 86.10 | Level 2 |
| M68 | 18.49 | PG(i-27:0) | C_33_H_65_O_10_P | 675.4214 | 3.55 | [M+Na]^+^ | 423.39, 255.23 | Level 2 |
| M69 | 18.59 | PC(34:1-O) | C_42_H_80_NO_9_P | 796.5469 | -9.42 | [M+Na]^+^ | 184.07, 125.00, 104.11, 86.09 | Level 2 |
| M70 | 18.67 | SM(d38:1) | C_43_H_87_N_2_O_6_P | 759.638 | 3.16 | [M+H]^+^ | 765.65, 706.58, 576.58 | Level 2 |
| M71 | 18.69 | PC(34:3-OH) | C_42_H_78_NO_9_P | 772.5492 | -9.06 | [M+H]^+^ | 184.07, 125.00, 104.10, 86.10 | Level 2 |
| M72 | 18.71 | PS(36:1-2OH) | C_42_H_80_NO_12_P | 822.5496 | 9.97 | [M+H]^+^ | 717.52, 637.54 | Level 2 |
| M73 | 18.73 | Glycerol 1,3-dihexadecanoate | C_35_H_68_O_5_ | 591.4965 | -8.79 | [M+Na]^+^ | 427.34, 401.32, 313.28, 299.25 | Level 2 |
| M74 | 18.74 | PS(38:2) | C_44_H_82_NO_10_P | 816.5755 | 1.47 | [M+H]^+^ | 711.55, 631.58 | Level 2 |
| M75 | 18.76 | PE(42:6) | C_47_H_82_NO_8_P | 842.5676 | -0.47 | [M+Na]^+^ | 184.07, 125.00, 104.10, 86.10 | Level 2 |
| M76 | 18.84 | PC(36:3-O) | C_44_H_80_NO_9_P | 820.5469 | -1.10 | [M+Na]^+^ | 184.07, 125.00, 104.10, 86.10 | Level 2 |
| M77 | 18.93 | PG(a-27:0) | C_33_H_65_O_10_P | 675.4214 | 3.55 | [M+Na]^+^ | 423.38, 255.23 | Level 2 |
| M78 | 19.21 | MG(i-22:0) | C_25_H_50_O_4_ | 437.3607 | 8.00 | [M+Na]^+^ | 432.40, 396.37, 341.34 | Level 2 |
| M79 | 19.29 | SM(d18:1/22:0) | C_45_H_91_N_2_O_6_P | 787.6693 | 8.76 | [M+H]^+^ | 809.65, 750.58, 586.60 | Level 2 |
| M80 | 19.35 | PC(O-34:3) | C_42_H_80_NO_7_P | 742.5751 | 7.81 | [M+H]^+^ | 184.08, 125.00, 104.11, 86.10 | Level 2 |
| M81 | 19.42 | SM(d18:0/22:1) | C_44_H_86_NO_8_P | 788.6169 | -5.45 | [M+H]^+^ | 727.56, 647.60 | Level 2 |
| M82 | 19.49 | Glyceryl behenate | C_25_H_50_O_4_ | 437.3607 | -1.83 | [M+Na]^+^ | 432.40, 415.39 | Level 2 |
| M83 | 19.59 | PG(i-29:0) | C_35_H_69_O_10_P | 703.4527 | 0.14 | [M+Na]^+^ | 453.43, 255.23 | Level 2 |
| M84 | 19.62 | DG(32:0) | C_35_H_68_O_5_ | 591.4965 | -0.34 | [M+Na]^+^ | 239.24, 237.22, 199.24, 145.09 | Level 2 |
| M85 | 19.67 | PC(38:4) | C_46_H_84_NO_8_P | 832.5833 | 6.25 | [M+Na]^+^ | 184.07, 125.00, 104.10, 86.10 | Level 2 |
| M86 | 19.69 | DG(34:0) | C_37_H_72_O_5_ | 619.5278 | -3.23 | [M+Na]^+^ | 619.52, 391.32, 307.23, 251.20 | Level 2 |
| M87 | 19.72 | alpha-Tocopherol succinate | C_33_H_54_O_5_ | 553.3869 | 9.58 | [M+Na]^+^ | 361.31, 319.27, 277.21, 207.14 | Level 2 |
| M88 | 19.74 | SM(d42:3) | C_47_H_91_N_2_O_6_P | 811.6693 | -3.45 | [M+H]^+^ | 817.68, 758.61, 628.60 | Level 2 |
| M89 | 19.76 | PC(36:1) | C_44_H_86_NO_8_P | 810.5989 | 1.36 | [M+Na]^+^ | 184.07, 125.00, 86.10 | Level 2 |
| M90 | 19.82 | DG(i-32:0) | C_35_H_68_O_5_ | 591.4965 | -8.79 | [M+Na]^+^ | 387.31, 317.30, 313.27, 299.26 | Level 2 |

MSI: Metabolomics Standards Initiative. Level 2: Putatively Annotated Metabolites (Putative Annotation); Level 3: Putatively Characterized Compound Class.

**Table S6.** The chemical classification and·quantitative information of differential metabolites.

| **No.** | **Class** | **VIP** | **FC** | ***p value*** | **No.** | **Class** | **VIP** | **FC** | ***p value*** |
| --- | --- | --- | --- | --- | --- | --- | --- | --- | --- |
| M1 | Prenol Lipids | 1.20 | 2.21 | ***** | M46 | Sterol Lipids | 1.13 | 0.81 | * |
| M2 | Sterol Lipids | 1.29 | 2.50 | ** | M47 | Sphingolipids | 1.55 | 1.29 | * |
| M3 | Others | 1.33 | 1.12 | * | M48 | Sterol Lipids | 1.31 | 0.31 | *** |
| M4 | Prenol Lipids | 1.15 | 0.46 | *** | M49 | Glycerolipids | 1.30 | 1.58 | * |
| M5 | Fatty Acyls | 1.42 | 2.70 | *** | M50 | Glycerophospholipids | 1.09 | 0.65 | ** |
| M6 | Sterol Lipids | 1.22 | 4.24 | *** | M51 | Glycerolipids | 1.17 | 1.86 | * |
| M7 | Prenol Lipids | 1.29 | 2.93 | *** | M52 | Prenol Lipids | 1.33 | 8.18 | *** |
| M8 | Others | 1.58 | 2.62 | *** | M53 | Fatty Acyls | 1.06 | 0.27 | *** |
| M9 | Fatty Acyls | 1.10 | 0.65 | ** | M54 | Glycerophospholipids | 1.14 | 0.51 | ** |
| M10 | Sphingolipids | 1.41 | 1.87 | *** | M55 | Glycerolipids | 1.55 | 1.84 | *** |
| M11 | Others | 1.27 | 0.65 | *** | M56 | Fatty Acyls | 1.60 | 1.44 | *** |
| M12 | Others | 1.19 | 0.70 | ** | M57 | Glycerolipids | 1.20 | 11.12 | *** |
| M13 | Others | 1.39 | 0.72 | *** | M58 | Glycerophospholipids | 1.13 | 1.93 | * |
| M14 | Others | 1.77 | 0.32 | *** | M59 | Glycerophospholipids | 1.63 | 107.03 | *** |
| M15 | Prenol Lipids | 1.22 | 0.72 | *** | M60 | Fatty Acyls | 1.10 | 1.44 | * |
| M16 | Sterol Lipids | 1.16 | 0.72 | * | M61 | Sterol Lipids | 1.00 | 7.97 | *** |
| M17 | Fatty Acyls | 1.12 | 5.80 | *** | M62 | Glycerophospholipids | 1.87 | 1.98 | *** |
| M18 | Glycerolipids | 1.15 | 1.90 | *** | M63 | Glycerolipids | 1.79 | 0.56 | *** |
| M19 | Fatty Acyls | 1.04 | 0.15 | *** | M64 | Glycerophospholipids | 1.04 | 1.43 | * |
| M20 | Fatty Acyls | 1.22 | 0.75 | ** | M65 | Others | 1.40 | 3.86 | *** |
| M21 | Glycerolipids | 1.29 | 1.94 | *** | M66 | Glycerolipids | 1.76 | 1.82 | *** |
| M22 | Fatty Acyls | 1.13 | 0.63 | * | M67 | Glycerophospholipids | 1.77 | 1.81 | ** |
| M23 | Glycerolipids | 1.11 | 1.86 | *** | M68 | Glycerophospholipids | 1.06 | 0.81 | *** |
| M24 | Glycerolipids | 1.37 | 3.91 | *** | M69 | Glycerophospholipids | 1.12 | 0.19 | *** |
| M25 | Others | 1.77 | 19.34 | *** | M70 | Sphingolipids | 1.23 | 3.00 | *** |
| M26 | Glycerophospholipids | 1.23 | 0.27 | *** | M71 | Glycerophospholipids | 1.14 | 2.31 | * |
| M27 | Glycerophospholipids | 1.52 | 1.29 | ** | M72 | Glycerophospholipids | 1.23 | 5.53 | *** |
| M28 | Glycerophospholipids | 1.42 | 2.22 | ** | M73 | Glycerolipids | 1.69 | 1.80 | *** |
| M29 | Glycerophospholipids | 1.63 | 13.10 | *** | M74 | Glycerophospholipids | 1.16 | 2.86 | ** |
| M30 | Glycerophospholipids | 1.37 | 1.93 | * | M75 | Glycerophospholipids | 1.19 | 0.22 | *** |
| M31 | Glycerolipids | 1.69 | 1.43 | ** | M76 | Glycerophospholipids | 1.02 | 0.33 | *** |
| M32 | Sterol Lipids | 1.79 | 7.43 | *** | M77 | Glycerophospholipids | 1.28 | 0.89 | *** |
| M33 | Glycerolipids | 1.00 | 1.71 | *** | M78 | Glycerolipids | 1.64 | 2.93 | *** |
| M34 | Glycerophospholipids | 1.43 | 2.66 | *** | M79 | Sphingolipids | 1.02 | 2.04 | * |
| M35 | Sphingolipids | 1.27 | 3.58 | *** | M80 | Glycerophospholipids | 1.50 | 2.75 | ** |
| M36 | Fatty Acyls | 1.31 | 0.76 | ** | M81 | Sphingolipids | 1.47 | 2.65 | *** |
| M37 | Prenol Lipids | 1.30 | 0.39 | *** | M82 | Glycerolipids | 1.15 | 1.86 | *** |
| M38 | Others | 1.43 | 0.32 | *** | M83 | Glycerophospholipids | 1.02 | 0.66 | *** |
| M39 | Sphingolipids | 1.52 | 1.56 | *** | M84 | Glycerolipids | 1.43 | 1.81 | *** |
| M40 | Glycerophospholipids | 1.65 | 3.14 | ** | M85 | Glycerophospholipids | 1.44 | 2.76 | *** |
| M41 | Sphingolipids | 1.56 | 1.88 | *** | M86 | Glycerolipids | 1.68 | 1.85 | * |
| M42 | Glycerophospholipids | 1.59 | 1.93 | *** | M87 | Prenol Lipids | 1.09 | 9.96 | *** |
| M43 | Glycerophospholipids | 1.63 | 2.13 | ** | M88 | Sphingolipids | 1.43 | 0.16 | *** |
| M44 | Glycerophospholipids | 1.60 | 2.15 | *** | M89 | Glycerophospholipids | 1.43 | 4.26 | *** |
| M45 | Glycerolipids | 1.18 | 1.51 | ** | M90 | Glycerolipids | 1.65 | 1.81 | *** |

* *p* < 0.05, ** *p* < 0.01, *** *p* < 0.001. “-” indicated that the metabolite was not among the differentially accumulated metabolites in this comparative analysis.

**Table S7.** Fold change data of 71 time-independent nonalcoholic fatty liver disease differential metabolites.

| **No.** | **Fold change** | | | |
| --- | --- | --- | --- | --- |
|  | **ND-3/ND-HFD-0** | **ND-6/ND-3** | **ND-9/ND-6** | **ND-12/ND-9** |
| M1 | 0.92 | 0.88 | 0.92 | 0.82 |
| M2 | 0.87 | 1.37 | 0.87 | 0.85 |
| M3 | 1.10 | 0.49 | 0.34 | 1.00 |
| M4 | 0.56 | 0.54 | 0.82 | 0.63 |
| M6 | 0.63 | 1.30 | 1.03 | 0.71 |
| M7 | 1.06 | 1.11 | 0.91 | 0.77 |
| M8 | 1.16 | 0.90 | 0.98 | 0.79 |
| M9 | 1.72 | 0.93 | 1.33 | 1.00 |
| M10 | 0.44 | 0.97 | 1.05 | 0.87 |
| M12 | 0.91 | 1.36 | 1.25 | 0.81 |
| M13 | 0.73 | 0.35 | 0.30 | 1.20 |
| M14 | 0.73 | 0.35 | 0.22 | 0.68 |
| M15 | 0.58 | 0.93 | 1.45 | 0.83 |
| M16 | 0.64 | 0.83 | 0.89 | 1.08 |
| M17 | 1.00 | 0.55 | 1.05 | 0.21 |
| M18 | 0.87 | 0.97 | 1.03 | 0.81 |
| M19 | 0.57 | 0.36 | 0.26 | 0.69 |
| M20 | 0.64 | 0.83 | 0.89 | 1.00 |
| M21 | 0.74 | 0.97 | 0.97 | 0.81 |
| M23 | 0.83 | 1.02 | 0.92 | 0.86 |
| M24 | 0.21 | 1.05 | 0.94 | 0.83 |
| M25 | 0.94 | 1.13 | 0.93 | 1.09 |
| M27 | 1.19 | 1.03 | 1.03 | 0.86 |
| M31 | 1.01 | 0.95 | 0.99 | 0.92 |
| M32 | 0.64 | 0.83 | 0.89 | 0.56 |
| M33 | 0.42 | 1.08 | 0.88 | 0.80 |
| M34 | 0.86 | 1.00 | 0.81 | 1.00 |
| M35 | 0.08 | 1.16 | 1.24 | 0.75 |
| M36 | 0.91 | 1.04 | 1.20 | 0.99 |
| M37 | 0.99 | 0.75 | 1.00 | 0.76 |
| M38 | 1.10 | 0.81 | 0.83 | 0.88 |
| M39 | 0.78 | 0.98 | 0.90 | 0.79 |
| M40 | 0.90 | 1.22 | 1.46 | 1.00 |
| M41 | 0.17 | 1.07 | 1.08 | 0.79 |
| M45 | 1.03 | 0.96 | 0.87 | 0.98 |
| M46 | 0.56 | 1.10 | 0.83 | 0.95 |
| M47 | 1.30 | 0.77 | 1.09 | 1.04 |
| M49 | 0.92 | 0.97 | 0.98 | 0.99 |
| M51 | 0.70 | 1.45 | 0.96 | 0.72 |
| M52 | 0.90 | 1.00 | 0.89 | 0.73 |
| M53 | 0.86 | 0.71 | 0.78 | 0.80 |
| M54 | 1.44 | 1.39 | 0.66 | 1.08 |
| M55 | 0.81 | 1.04 | 0.97 | 0.75 |
| M56 | 0.91 | 0.79 | 0.80 | 0.71 |
| M59 | 0.52 | 0.63 | 0.63 | 0.05 |
| M60 | 1.00 | 0.99 | 0.98 | 0.92 |
| M61 | 0.20 | 1.13 | 1.03 | 0.99 |
| M63 | 0.65 | 0.61 | 1.22 | 1.49 |
| M64 | 0.92 | 0.91 | 0.97 | 0.74 |
| M65 | 1.01 | 0.87 | 0.81 | 0.95 |
| M66 | 0.94 | 1.08 | 0.84 | 0.82 |
| M67 | 0.81 | 0.96 | 0.94 | 0.95 |
| M68 | 0.96 | 0.95 | 0.97 | 0.91 |
| M69 | 2.11 | 1.00 | 1.82 | 0.95 |
| M70 | 0.62 | 0.63 | 0.81 | 1.02 |
| M72 | 0.79 | 0.96 | 0.88 | 0.73 |
| M73 | 0.70 | 1.19 | 1.04 | 0.71 |
| M74 | 0.10 | 0.47 | 1.24 | 0.64 |
| M76 | 1.24 | 1.11 | 0.99 | 0.97 |
| M77 | 0.90 | 0.96 | 1.10 | 0.83 |
| M78 | 0.56 | 1.12 | 0.99 | 0.61 |
| M80 | 0.83 | 0.51 | 1.28 | 0.65 |
| M81 | 0.50 | 0.53 | 0.94 | 1.56 |
| M82 | 0.94 | 0.93 | 0.82 | 0.94 |
| M83 | 1.06 | 0.93 | 1.05 | 0.88 |
| M84 | 0.70 | 1.19 | 1.05 | 0.71 |
| M86 | 0.70 | 1.44 | 0.96 | 0.72 |
| M87 | 1.09 | 1.13 | 0.99 | 0.86 |
| M88 | 0.56 | 0.65 | 0.70 | 0.23 |
| M89 | 0.53 | 0.57 | 0.99 | 0.63 |
| M90 | 0.98 | 0.94 | 0.83 | 0.93 |

“ND-x” indicate mice at week x of the normal diet. “ND-HFD0” is the baseline group.

**Table S8.** Related trend clustering and correlation information of biomarkers.

| No. | Cluster | |  | Correlation analysis | |
| --- | --- | --- | --- | --- | --- |
|  | HFD | ND |  | *r* | *p* value |
| M2 | 4 | 2 |  | 0.76 | * |
| M6 | 4 | 2 |  | 0.85 | ** |
| M7 | 4 | 2 |  | 0.78 | * |
| M8 | 4 | 3 |  | 0.76 | * |
| M18 | 4 | 1 |  | 0.83 | ** |
| M23 | 3 | 1 |  | 0.75 | * |
| M25 | 4 | 5 |  | 0.87 | ** |
| M27 | 5 | 5 |  | 0.82 | ** |
| M31 | 3 | 3 |  | 0.83 | ** |
| M32 | 4 | 1 |  | 0.75 | * |
| M34 | 3 | 1 |  | 0.84 | ** |
| M40 | 5 | 5 |  | 0.96 | *** |
| M45 | 5 | 3 |  | 0.78 | * |
| M47 | 5 | 5 |  | 0.82 | ** |
| M49 | 3 | 1 |  | 0.88 | ** |
| M51 | 5 | 2 |  | 0.73 | * |
| M52 | 4 | 3 |  | 0.85 | ** |
| M60 | 3 | 3 |  | 0.73 | * |
| M65 | 4 | 3 |  | 0.75 | * |
| M67 | 5 | 1 |  | 0.83 | ** |
| M72 | 5 | 1 |  | 0.88 | ** |
| M76 | 5 | 5 |  | 0.92 | *** |
| M82 | 3 | 3 |  | 0.68 | * |
| M86 | 5 | 2 |  | 0.73 | * |
| M87 | 4 | 2 |  | 0.86 | ** |
| M90 | 3 | 3 |  | 0.70 | * |

“ND” denotes mice fed a normal diet; “HFD” denotes mice fed a high-fat diet. Under HFD conditions, Clusters 3 and 5 represent gradual accumulation patterns, while Cluster 4 corresponds to a mid-phase surge pattern. Under ND conditions, Clusters 1 and 3 indicate gradual attenuation patterns, whereas Clusters 2 and 5 reflect stable maintenance patterns. “*” indicates *p* < 0.05, “**” indicates *p* < 0.01, and “***” indicates *p* < 0.001.

**Table S9.** Quantitative and correlation information of 22 biomarkers.

| **No.** | **NC vs. MM** | |  | **CI vs. MM** | |  | **Correlation analysis** | |
| --- | --- | --- | --- | --- | --- | --- | --- | --- |
|  | **FC** | ***p* value** |  | **FC** | ***p* value** |  | ***r*** | ***p* value** |
| M2 | 2.23 | *** |  | 0.43 | ** |  | 0.64 | *** |
| M6 | 4.82 | *** |  | 0.42 | ** |  | 0.79 | *** |
| M18 | 1.79 | *** |  | 0.84 | ** |  | 0.74 | *** |
| M23 | 1.78 | *** |  | 0.85 | *** |  | 0.80 | *** |
| M27 | 1.29 | *** |  | 0.83 | * |  | 0.91 | *** |
| M31 | 1.39 | *** |  | 0.87 | * |  | 0.62 | ** |
| M32 | 7.50 | *** |  | 0.26 | ** |  | 0.91 | *** |
| M34 | 3.53 | *** |  | 0.56 | ** |  | 0.80 | *** |
| M40 | 2.85 | *** |  | 0.47 | ** |  | 0.85 | *** |
| M45 | 2.54 | *** |  | 0.46 | *** |  | 0.61 | ** |
| M47 | 1.27 | *** |  | 0.80 | * |  | 0.60 | * |
| M49 | 1.79 | *** |  | 0.72 | ** |  | 0.78 | *** |
| M51 | 2.38 | *** |  | 0.49 | *** |  | 0.78 | *** |
| M52 | 9.94 | *** |  | 0.16 | *** |  | 0.93 | *** |
| M60 | 1.68 | *** |  | 0.64 | ** |  | 0.88 | *** |
| M67 | 17.66 | *** |  | 0.07 | *** |  | 0.60 | * |
| M72 | 3.60 | *** |  | 0.23 | *** |  | 0.64 | ** |
| M76 | 2.40 | *** |  | 0.74 | * |  | 0.71 | *** |
| M82 | 1.98 | *** |  | 0.88 | * |  | 0.73 | *** |
| M86 | 1.78 | ** |  | 0.34 | *** |  | 0.65 | *** |
| M87 | 9.44 | *** |  | 0.10 | *** |  | 0.93 | *** |
| M90 | 2.12 | *** |  | 0.77 | ** |  | 0.66 | *** |

NC: normal control; MM: MASLD model; CI: curcuminoids intervention; FC: fold change. **p* < 0.05, ***p* < 0.01, ****p* < 0.001.
